# Causal Relationship Between Serum Uric Acid and Atherosclerotic Disease: A Mendelian Randomization and Transcriptomic Analysis

**DOI:** 10.3390/biomedicines13081838

**Published:** 2025-07-28

**Authors:** Shitao Wang, Shuai Mei, Xiaozhu Ma, Qidamugai Wuyun, Li Zhou, Qiushi Luo, Ziyang Cai, Jiangtao Yan

**Affiliations:** 1 Division of Cardiology, Departments of Internal Medicine, Tongji Hospital, Tongji Medical College, Huazhong University of Science and Technology, Wuhan 430030, China; u202010365@hust.edu.cn (S.W.);; 2 Hubei Key Laboratory of Genetics and Molecular Mechanisms of Cardiological Disorders, Wuhan 430030, China

**Keywords:** uric acid, coronary heart disease, mendelian randomization, transcriptomics

## Abstract

**Background/Objectives**: Elevated serum uric acid levels are associated with the occurrence, development, and adverse events of coronary heart disease (CHD) and CHD risk factors. However, the extent of any pathogenic effect of the serum uric acid on CHD and whether CHD risk factors play a confounding or mediating role are still unclear. **Methods**: The potential causal associations of serum uric acid with CHD were evaluated via cross-trait linkage disequilibrium score regression analysis and Mendelian randomization. The pleiotropy of genetic tools was analyzed via a Bayesian colocalization approach. Moreover, we utilized two-step MR to identify risk factors mediating the relationship between uric acid and CHD. **Results**: Mendelian randomization results derived from two genetic instrument selection strategies support that serum uric acid levels have a significant causal relationship with coronary artery disease, stable angina pectoris, and myocardial infarction. This causal relationship was partially mediated by diastolic blood pressure, mean arterial pressure, and serum triglycerides. Transcriptomic analysis revealed that serum uric acid may directly contribute to the development of atherosclerosis by inducing transcriptomic changes in macrophages. **Conclusions**: Our findings highlight that the control of serum urate concentration in the long-term management of CHD patients may be necessary. Well-designed clinical trials and foundational research are presently required to furnish conclusive proof regarding the specific clinical scenarios in which adequate reduction in urate concentrations can confer cardiovascular advantages.

## 1. Introduction

In recent decades, the dramatic changes in people’s lifestyle and diet structure led to the widespread prevalence of diabetes and obesity, thereby increasing the burden of cardiovascular disease. The prevalence of hyperuricaemia, a metabolic disease, is also high and increasing. Up to the year 2016, the prevalence of hyperuricaemia in the United States adults reached 20% [1]. Two large-scale cross-sectional surveys of the Chinese adult population revealed that the prevalence of hyperuricaemia in the Chinese population increased from 11.1% to 14.0% [2]. Although it is increasingly accepted that high serum uric acid levels may be a potential risk factor for cardiovascular disease [3,4,5,6], the causality between them remains undetermined. Defining the relationship between hyperuricaemia and coronary heart disease (CHD) may be important for reducing the cardiovascular disease burden.

Previous observational studies revealed that increased serum urate is independently associated with coronary plaque formation [7], coronary calcification [8], the degree of coronary stenosis [9,10], and the number of coronary artery vasculopathy [10,11] after adjusting for CHD risk factors, which suggests that hyperuricaemia may be a potential pathogenic factor for CHD. In addition, hyperuricaemia is also considered to be associated with adverse cardiovascular events of CHD, such as recurrent nonfatal myocardial infarction [10], percutaneous coronary intervention or coronary artery bypass grafting [10,12], acute coronary syndrome (ACS) [13] and stroke [14]. Observational studies reported the protective effects of urate-lowering therapy on CHD, such as reducing the risk of MI [15,16]. However, the findings of observational studies are highly susceptible to reverse causality and confounding factors, and some observational studies about uric acid-lowering therapy are not consistent with the above results [17,18]. Whether a causality exists between serum urate and CHD progression, adverse events, and CHD risk factors remains unclear. Although a Mendelian randomization study suggested a potential causal relationship between serum uric acid levels and coronary heart disease and myocardial infarction [19], the study involved a limited number of subtypes of CHD, did not adjust for CHD risk factors, and did not reveal an independent causal relationship between serum uric acid and coronary heart disease.

In population-based studies, elevated serum uric acid is associated with multiple risk factors for CHD, such as hypertension [16,20,21,22,23], type II diabetes [24], dyslipidemia [25], insulin resistance [26], and metabolic syndrome [27]. However, whether these risk factors mediate or confound the causality of uric acid with CHD is obscure. It remains unknown whether these confounders can lead to an overestimation of the association or whether it is underestimated because some risk factors are mediators. In another MR study, blood pressure partially mediated the causal relationship between serum uric acid and CHD [28], but this study did not involve other CHD risk factors.

There are many potential biases in observational epidemiological studies, including confounding and reverse causality, which limits their ability to identify causality. In this research, we used Mendelian randomization (MR) [29] to estimate the extent of causality between serum uric acid and CHD, stable angina pectoris (SAP), unstable angina pectoris (UAP), myocardial infarction (MI), and ischemic stroke, and to determine whether CHD risk factors, such as LDL cholesterol, total cholesterol, triglycerides, hypertension, pulse pressure, diastolic pressure, systolic pressure, mean arterial pressure, and type II diabetes play a mediating role in it. Mendelian randomization (MR) analysis takes advantage of the random assignment of genetic variation at gametogenesis, which leaves the association of genotype with phenotype not affected by a variety of environmental factors and confounding sources in observational studies. Furthermore, the genotype of an individual after zygote formation is stable and constant, which makes MR studies less susceptible to reverse causality. In the context of MR, the allocation of genetic variants at conception, which serves as a predictor for a specific phenotype, mimics the random assignment of a treatment for that phenotype in a randomized controlled trial. When certain specific assumptions are satisfied, if genetic variation is associated with both biomarkers (such as urate) and outcome (such as CHD), this would support a causal effect of the biomarkers on the outcome [29].

In this study, we employed a two-sample MR approach to investigate the causal relationships between serum urate concentrations and the occurrence, progression, and adverse events of CHD. We conducted a comprehensive scan of the GWAScatlog database and performed Bayesian colocalization analysis to elucidate the pleiotropy present in the genetic instruments. Multivariable Mendelian randomization (MVMR) was utilized to adjust for known pleiotropic effects. To ensure the robustness of our findings, we replicated the analyses using a biology-driven genetic instrument selection strategy. Additionally, a two-step MR method was applied to identify whether CHD risk factors mediated these causal associations. Finally, we explored the underlying biological mechanisms through transcriptome analysis.

## 2. Materials and Methods

All the data utilized in this study are either publicly available or can be accessed through the IEU openGWAS datasets [30,31], GWAS catalog, and the GEO database. SNP-level summary data for MR were extracted online from the ieu openGWAS database via the TwoSampleMR package (version 0.6.2). SNP-level summary data for colocalization and linkage disequilibrium regression analyses were downloaded from the ieu openGWAS database and GWAS catalog database.

### 2.1. Study Design

**Figure 1:**
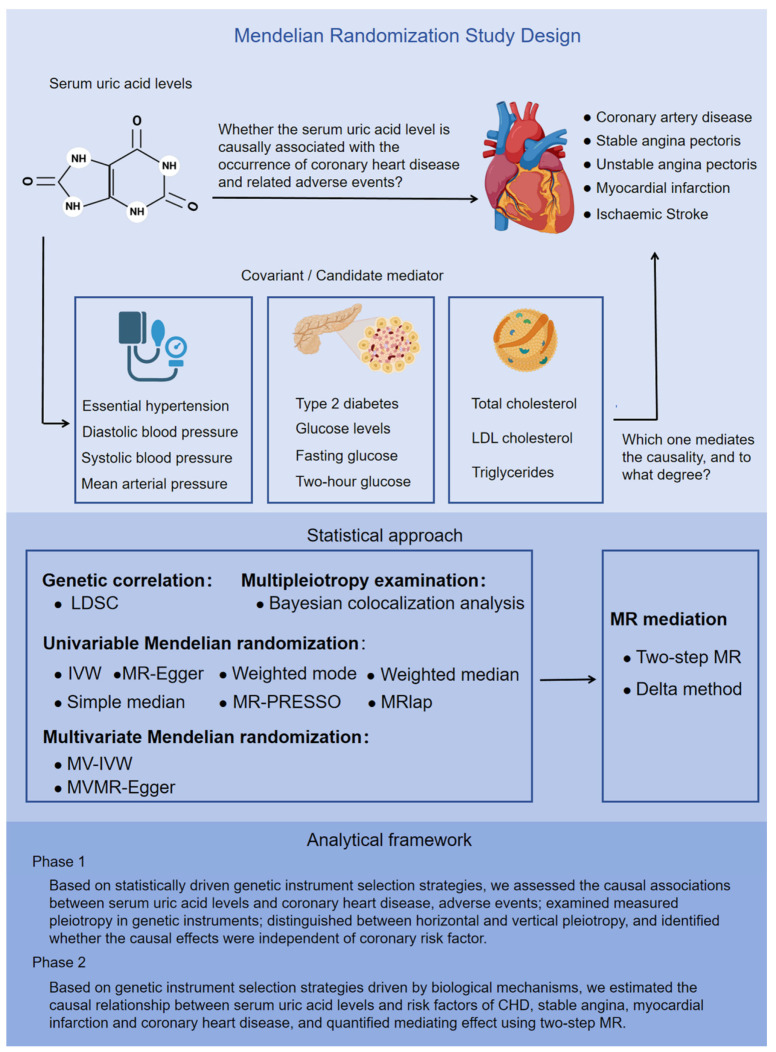
**Overview of the Mendelian randomization study design.** This MR study comprised two analysis phases. In phase 1, on the basis of statistically driven genetic instrument selection strategies, we assessed the causal associations between serum uric acid levels and the occurrence, development, and adverse events of CHD; and identified whether the causal effects were independent of coronary risk factors via UVMR and MVMR. In phase 2, on the basis of genetic instrument selection strategies driven by biological mechanisms, we estimated the causal relationships between serum uric acid levels and risk factors for CHD, stable angina, myocardial infarction, and coronary heart disease, and quantified the mediating effects via two-step MR.

Our study employs a two-sample MR approach, utilizing summary data at the single-nucleotide polymorphisms (SNPs) level. This MR (MR) investigation comprised two distinct analytical stages (Figure 1). In stage 1, our analysis was grounded in statistically driven genetic instrument selection strategies. Specifically, we conducted the following tasks: (1) Causality evaluation: we evaluated the causal relationship between serum urate and the occurrence, development, as well as adverse events associated with CHD. (2) Pleiotropy analysis: we analyzed the pleiotropic effects of the genetic instruments used in our study. (3) Pleiotropy classification: we differentiated between horizontal and vertical pleiotropy among these genetic instruments. (4) Independence verification: using univariate Mendelian randomization (UVMR) and MVMR, we determined whether the observed causality was independent of known risk factors for CHD.

In stage 2, based on genetic instrument selection strategies driven by biological mechanisms, we estimated the causality between serum urate and risk factors for CHD, SAP, and MI, and quantified the mediating effect via two-step MR. We used datasets from independent populations to validate our MR results both in stage 1 and stage 2. These validation datasets were derived from the CARDIoGRAMplusC4D study, FinnGen Biobank, and the VA Million Veteran Program. Supplementary Table S1 provides a comprehensive overview of the GWAS data employed in this study. Furthermore, we examined the transcriptomic to elucidate how urate contributes to the progression of atherosclerotic lesions. This study adhered to the reporting guidelines outlined in Strengthening the Reporting of Observational Studies in Epidemiology using Mendelian randomization (STROBE-MR) [32]. Supplementary Table S1 provides the STROBE-MR checklist. This research leveraged publicly accessible summary-level GWAS data, allowing us to reference the ethical approvals of the original GWAS studies in the publications cited herein.

### 2.2. Data Resources

The published meta-GWAS provided summary-level GWAS data on serum urate concentrations in European populations [33]. This study used the UKB biobank and FinnGen biobank to obtain genome-wide significant association loci for serum urate concentrations in Europeans. The genotypes in the study underwent imputation using a combination of reference panels from the phase 3 of the 1000 Genomes Project, UK10K, and Haplotype Reference Consortium. Therefore, this study is currently the largest sample size and detection of SNPs among genome-wide association studies on serum urate concentrations. To ensure that SNP sites in the exposure could be available in the outcome data, we prioritized the SNP number measurement in GWASs when selecting GWAS data. Supplementary Table S2 provides a comprehensive overview of the GWAS data employed in this study. The original study contains a detailed elaboration on the selection criteria, sources, and methodologies for participant recruitment in each GWAS, alongside measures pertaining to genetic variation, quality control procedures, and selection methodologies.

Overview of the transcriptomic data. GSE107821: Transcriptome of human monocytes subjected to in vitro urate treatment. Monocytes derived from healthy donors were cultured in the context of uric acid exposure for 24 h, after which the cells were harvested for RNA-seq in four biological replicates. GSE155513: Ldlr-/-mice were subjected to single-cell sequencing of arterial tissue (including the ascending artery, brachiocephalic artery, and thoracic aorta) at four time points (0, 8, 16, and 26 weeks) after Western diet (WD) feeding.

### 2.3. Genetic Instrument Selection

A statistically driven genetic instrument selection strategy was used. Each genetic variant demonstrated a statistically significant association with serum urate concentrations at the genome-wide significance threshold (*p* < 5.00 × 10^−8^) and was independent of each other [linkage disequilibrium (LD) r^2^ < 0.001 within 10,000 kb]. In cases where SNPs related to the exposure were absent from the summary statistics of outcomes, proxy SNPs were utilized as substitutes. The proxy SNPs were identified based on a high degree of LD, specifically with an r^2^ value exceeding 0.8 via the 1000 Genomes Reference Panel from European samples. The advantage of this strategy is that more genetic variants can be obtained as instrumental variables, improving the statistical power of MR. However, the bias caused by the pleiotropy of genetic instruments cannot be avoided.

A biologically driven genetic instrument selection strategy was used. We conducted a search for protein-coding genes implicated in the metabolism and transportation of urate utilizing the PANTHER classification system [34] and the relevant protein function support evidence in PubMed, and then selected protein-coding genes involved only in uric acid metabolism and transport to avoid possible genetic instrument pleiotropy problems. We retrieved SLC22A12, SLC2A9, ABCG2, SLC17A1, SLC22A11, SLC17A3, and SLC16A9, which are uric acid transporter proteins expressed mainly in the kidney or liver (Supplementary Table S3). We examined and selected SNPs located within the coding regions of these genes, which exhibit genome-wide significant associations with serum urate concentrations (*p* < 5 × 10^−8^) and are mutually independent, as indicated by a linkage disequilibrium (LD) r^2^ value less than 0.35. In the biologically driven genetic instrument selection strategy, considering the inter-variant correlations, the incorporation of variants displaying mild correlation can enhance statistical efficiency, as opposed to selecting solely those that are strictly independent [35]. However, MR estimators may exhibit instability when genetic variants are excessively correlated. To address this, a pruning threshold for the r^2^ value was determined to strike a balance between these competing concerns [36]. The advantage of this strategy is that the relativity of genetic instruments with serum urate is supported by biological mechanisms to avoid the effects of pleiotropy. However, owing to the difficulty in obtaining a sufficient amount of independent genetic variation, the statistical power of MR may be insufficient.

We calculated the overall phenotypic variance-explained R^2^ by summing the estimated R^2^[R = 2 × MAF × (1 − MAF) × beta^2^/(2 × beta^2^ × MAF(1 − MAF) + se^2^ × 2 × N × beta^2^ × MAF(1 − MAF))] [37] for each SNP. The F statistic [F = R^2^ × (N − 2)/(1 − R^2^)] for each SNP under consideration exceeded 30 [38] (Supplementary Tables S5 and S6), indicating no potential weak instrument bias.

### 2.4. Statistical Analysis

#### 2.4.1. Cross-Trait Linkage Disequilibrium Score Regression Analysis (LDSC)

Cross-trait LDSC [39,40] was used to assess the genetic correlations between serum urate concentrations and the occurrence, development, and adverse events of CHD and CHD risk factors. The LDSC method estimates genetic correlation by taking into account the contributions of all SNPs, irrespective of whether they attain genome-wide significance. LDSC facilitates the evaluation of genetic correlations between two traits, providing a comprehensive array of genetic insights while remaining unaffected by sample overlap. A notable limitation of this approach, however, is its inability to deduce causal relationships [39]. The results are presented in the form of genetic correlation (rg) accompanied by its corresponding standard error (SE). *p*-values below 0.05 were deemed indicative of potential genetic correlation. Statistical analyses were conducted utilizing the ldscr software version 1.0.1.

#### 2.4.2. UVMR and MVMR Analysis

MR analysis is a form of instrumental variable analysis that employs genetic variants as proxy instrumental variables (IVs) to investigate causality. MR analysis rests on three core assumptions: (1) the genetic instruments are associated with the exposure variable; (2) the genetic instruments are unconfounded by any extraneous factors; (3) the genetic instruments exert their influence on outcomes solely through the exposure.

For each UVMR analysis of the combination of exposure outcomes, we removed the genetic instruments that brought greater heterogeneity. The specific method is as follows: We measured the influence of the genetic instruments on the heterogeneity by testing the remaining genetic tools for the heterogeneity after excluding each SNP. Then, following this prioritization, the SNP was sequentially removed until a robust UVMR result with no significant heterogeneity was obtained. Supplementary Table S7 describes the SNP and rationtification for elimination in the UVMR analysis for each combination of exposure outcome (Supplementary Table S7).

All MR analyses were performed using the R software (version 4.3.1) with the aid of the R packages TwoSampleMR (version 0.6.2), MR-PRESSO (version 1.0), and MRlap (version 0.0.3.2). All statistical tests performed were bilateral, and statistical significance was deemed present when the *p* value was less than 0.05. The IVW estimates were deemed to represent causality only when they exhibited the same direction and statistical significance as at least one sensitivity analysis. For the causal association analyses, the false discovery rate (FDR) q values were computed using both the Benjamin–Hochberg and Bonferroni methods to address multiple hypothesis testing [41]. The MR estimates are presented as odds ratios (ORs), β coefficients, or proportions, along with their corresponding 95% confidence intervals (CIs).

#### 2.4.3. Bayesian Colocalization Analysis

To distinguish the horizontal and vertical pleiotropy of each SNP, we performed Bayesian colocalization via the coloc statistical approach, between the serum urate concentrations and all coronary risk factors for which a both statistically significant genome-wide associations were identified. A range of 100,000 bases upstream and downstream of each SNP site was selected for colocalization analysis. Coloc provides evidence for five potential scenarios or hypotheses: (1) there is no causal genetic variant associated with either trait (H0); (2) there is a single causal genetic variant exclusively for trait 1 (H1); (3) there is a single causal genetic variant exclusively for trait 2 (H2); (4) there are two separate causal genetic variants, one for each trait (H3); and (5) there is a shared causal genetic variant affecting both traits (H4). The support for each hypothesis is measured by the posterior probability (PP), denoted, respectively, as PPH0, PPH1, PPH2, PPH3, and PPH4 [42].

A posterior probability for PPH4 exceeding 0.75 provides strong evidence for the existence of a common causal genetic variant influencing both traits (which is also referred to as vertical pleiotropy). On the other hand, if the posterior probability for PPH3 surpasses 0.75, it indicates a high likelihood of distinct causal genetic variants underlying each trait, suggesting potential confounding due to linkage disequilibrium in the corresponding MR analysis. This is also referred to as horizontal pleiotropy. The presence of vertical pleiotropy does not violate the underlying assumption of MR. When both PPH3 and PPH4 are below 0.8, it implies that the colocalization analysis may lack sufficient power to differentiate whether the MR association arises from a shared causal variant or a variant in linkage disequilibrium that introduces confounding (i.e., horizontal pleiotropy).

Statistical analysis was performed via coloc package (version 5.2.1) in R software (version 4.3.1).

### 2.5. LDtrait

LDtrait [43], an accessible online resource located at https://ldlink.nih.gov/?tab=ldtrait (accessed on 20 October 2024), aids in identifying reported phenotypic associations that are in linkage disequilibrium. To comprehensively examine the possible known pleiotropy of the genetic instrument variables we used, we used the LDtrait online tool to identify traits or diseases associated with each SNP (R^2^ > 0.8, *p*-value < 5 × 10^−8^). This correlation type is due to linkage disequilibrium with genetic variants.

### 2.6. LDmatrix

We calculated a linkage disequilibrium matrix between SNPs from the biologically driven genetic instrument selection strategy via the LDmatrix online tool (https://ldlink.nih.gov/?tab=ldmatrix) (accessed on 29 October 2024) to determine whether r^2^ < 0.35 was met. In addition, the FORGEdb [44] scores were calculated for each genetic tool.

### 2.7. Transcriptome Difference Analysis and Enrichment Analysis

Differential gene analysis of the transcriptome was performed via the GEO2R tool from the GEO database, which performs differential gene analysis on the basis of DEseq2 [45]. The GSE107821 dataset has been standardized. To evaluate the potential biological roles of the differentially expressed genes (DEGs), gene ontology (GO), and Kyoto Encyclopedia of Genes and Genomes (KEGG) pathway enrichment analyses were conducted using Metascape [46]. The results of the enrichment analysis were visualized via the ggplot2 package in the R software (version 4.3.1).

### 2.8. Analysis of Mouse scRNA-Seq Data

The single-cell transcriptome dataset GSE155513 of arterial tissue from a mouse model of atherosclerosis for eight samples was downloaded from the GEO database by us. The datasets were analyzed via Seurat v5.0.2 in R software (version 4.3.1). Genes that were expressed in less than ten cells were omitted from subsequent analysis. We performed the following filtering process on the individual cells within each dataset: gene count, unique molecular identifier (UMI) count, and proportion of reads aligning to mitochondrial genes. These filtering parameters were all determined according to the original study (Supplementary Table S29). Subsequently, graphs illustrating various crucial metrics and their interrelationships were examined. These encompass the following: (i) two inflection point plots, with each cell ranked by total UMI counts and gene RNA counts, respectively, to detect the discontinuous plateau caused by empty droplets; (ii) a scatter plot displaying the correlation of UMI counts versus gene RNA counts in each cell, which is used to assess sequencing depth and outlier data; (iii) a scatter plot, which shows the correlation between the number of UMIs per cell and the fraction of mitochondrial RNA, which is used to assess whether there is an abnormal correlation; and (iv) density distribution plots of the ratio of mitochondrial RNA and the ribosomal RNA ratio in each sample, which are used to determine whether the distribution was concentrated and whether the distribution was significantly different in each sample. In addition, we also performed the quality control in the UMAP space. In the UMAP space, we consider different QC parameters to find the collective abnormal activity of a set of cells. This can reveal abnormalities that could not be detected in the first layer of quality control. For example, for a group of cells that simultaneously have a lower UMI count, a greater proportion of mitochondrial RNA and a greater proportion of ribosomal RNA may represent them as a population of cells in an apoptotic state.

The raw UMI counts for each cell were scaled by dividing by the total counts within that cell, multiplied by a factor of 10,000, and subsequently subjected to natural log transformation after adding a pseudocount of 1. Genes exhibiting an average expression level ranging from 0.05 to 10 and a dispersion value between 1.5 and 20 were chosen as those demonstrating high variability. To integrate datasets across time points and SMC lineages while minimizing batch effects, we used an integrated pipeline on Seurat built on a canonical correlation analysis (CCA). We performed this step via the IntegrateLayers function in Seurat v5.0.2. A two-dimensional UMAP was visualized via the top 20 principal components (PCs) established via the integrative analysis. Clustering analysis based on graph theory was applied to the combined dataset. For the Ldlr-/- mouse scRNA-seq data, a shared nearest neighbor (SNN) graph was established, utilizing 50 nearest neighbors and 20-dimensional principal components as input features. Clusters were identified with a resolution parameter of 0.4. We used the FindConservedMarkers function to identify cell type markers conserved under different experimental conditions. Through the use of the cell type markers identified by the original study and the PanglaoDB database [47], we manually annotated the cell types of each cell cluster.

### 2.9. High-Dimensional Weighted Gene Co-Expression Network Analysis (hdWGCNA)

We used the hdWGCNA workflow developed by Sam Morabito et al. [48], and gene coexpression networks were constructed from the single-cell transcriptome data. This analysis was performed via hdWGCNA (version 0.3.03) in R software (version 4.3.1). We constructed weighted gene coexpression networks for the three identified macrophage types. To reduce the sparsity of the single-cell expression matrix, the process first aggregates a small population of similar cells called meta cells. The mean expression of the genes was calculated to obtain the meta cell expression matrix. Owing to the small number of macrophages in each biological sample, we aggregated 12 cells with similar expression into one meta cell. Owing to differential gene expression among the three types of macrophages, we used different soft power thresholds (Supplementary Figure S6A). The connectivity of each gene in the module was calculated, and the top 20 genes for connectivity were selected as the hub genes of a module. The correlations of all the gene modules with the mold-making time were calculated for the three macrophage subtypes.

### 2.10. Code Availability

All packages for data analysis used in this study were open source in R software (version 4.3.1). All the MR analyses were conducted using R packages TwoSampleMR (version 0.6.2), MR-PRESSO (version 1.0), MRlap (version 0.0.3.2), coloc package (version 5.2.1), ldscr package (version 1.0.1), Seurat (version 5.0.2) and hdWGCNA (version 0.3.03) in R software (version 4.3.1). Custom code that supports the findings of this study is available at https://github.com/shitao2001/UA-AS (7 April 2025).

## 3. Results

### 3.1. The Causality Between Serum Urate Concentrations and CHD

**Figure 2:**
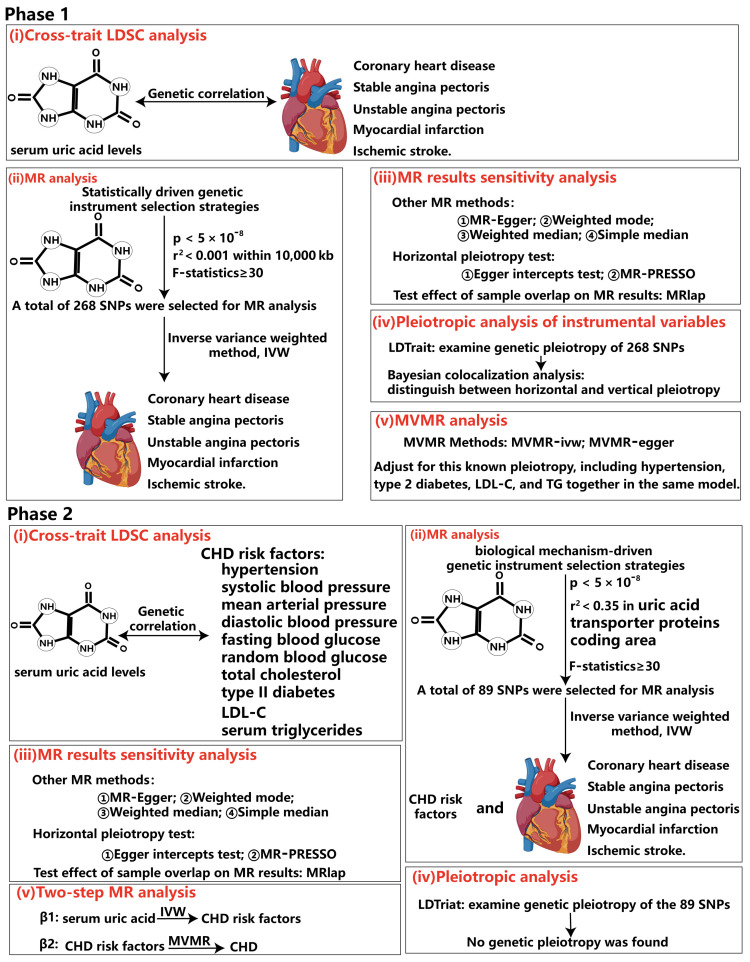
**Overview of the rigorous analytical paradigm for Mendelian randomization.** In phase 1, our analysis was grounded in statistically driven genetic instrument selection strategies. (**i**) We first calculated the genetic correlation between serum uric acid levels and five subtypes of coronary heart disease by cross-phenotype LDSC analysis. The results can support the reliability of the conclusions of subsequent MR analysis. (**ii**) Next, we used a statistics-driven genetic instrument selection strategy to identify 298 SNPs that were significantly associated with serum uric acid levels independently. MR analysis was performed using these SNPs to identify subtypes of CHD with potential causal relationship with serum uric acid levels. (**iii**) We then conducted sensitivity analyses to verify the robustness of the MR analysis conclusions. (**iv**) Subsequently, we employed LDtrait to examine genetic pleiotropy of the 298 identified SNPs, specifically assessing their significant correlation with CHD risk factors. Furthermore, Bayesian co-location analysis was employed to distinguish between horizontal and vertical epistatic interactions. (**v**) Finally, to correct for SNP genetic pleiotropy identified in step IV, we used multivariate Mendelian randomization to analyze the independent causation of serum uric acid levels and CHD subtypes. In phase 2, our analysis was grounded in biological mechanisms driven genetic instrument selection strategies. (**i**) Similar to phase 1, cross-phenotype LDSC was used to calculate the genetic correlation between serum uric acid levels and multiple CHD risk factors. (**ii**) Using biologically mechanism-driven genetic tool selection strategies, a total of 89 SNPs were identified. MR analysis validated the aforementioned MR conclusions while investigating potential causal relationships between serum uric acid levels and various CHD risk factors, preparing for subsequent mediation MR analysis. (**iii**) The same sensitivity analysis was conducted to test the robustness of the MR conclusions. (**iv**) LDtrait was similarly employed to examine SNP genetic epistasis. However, no significant correlations were found between these 89 SNPs and CHD risk factors. (**v**) Finally, mediation analysis was performed using a two-step MR method.

**Figure 3:**
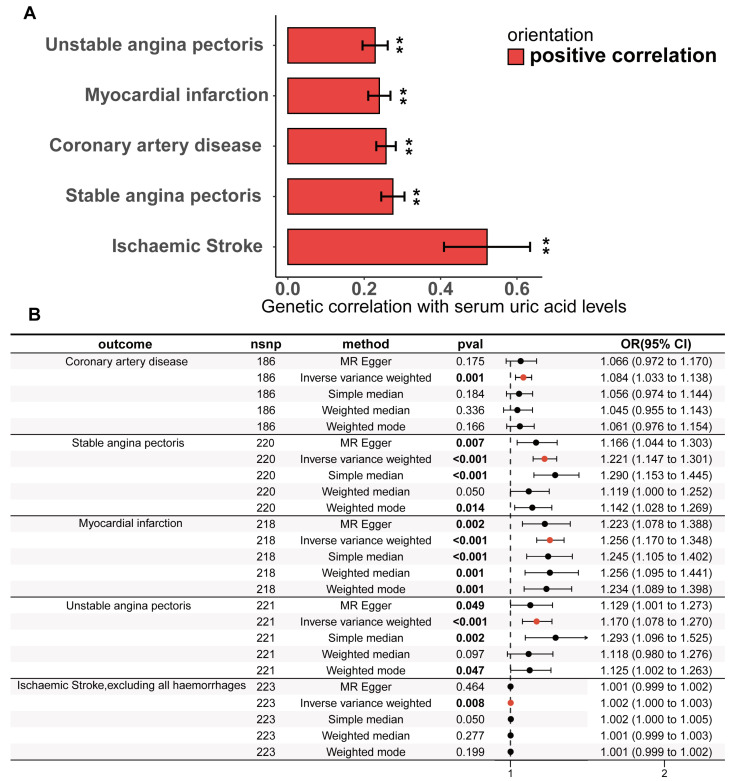
**Genetic correlation and causality estimates of serum uric acid levels and the occurrence of CHD and related adverse events**. (**A**) Genetic associations of serum uric acid levels with the occurrence, development, and adverse events of CHD were estimated via linkage disequilibrium regression analysis. (**B**) Causal effects of serum uric acid levels on CHD and related adverse events estimated by univariate two-sample Mendelian randomization on the basis of a statistically driven genetic tool selection strategy. Results with p values less than 0.05 are bolded. Values are shown as rg ± SE. ** indicates that the hypothesis test result is less than the *p*-value of significance calculated via the Bonferroni method. The LDSC data are presented as genetic correlation (rg) ± SE.

The study design is illustrated in Figure 1. The detailed research process of this study is shown in Figure 2. Cross-trait LDSC analysis unveiled genetic relativity between serum urate concentrations and conditions such as CHD, SAP, UAP, MI, and ischaemic stroke in the European population (range of absolute values of genetic correlation, |0.229| to |0.522|; *p* = 8.57 × 10^−24^ to 3.75 × 10^−6^; Figure 3A; Supplementary Table S4).

The statistically driven genetic tool selection strategy selected a total of 268 SNPs (Supplementary Table S5). Based on our initial IVW data analysis, after adjusting for multiple comparisons using the false discovery rate (FDR < 0.05), we observed positive causal links between serum urate concentrations and CHD (OR = 1.08, 95% CI = 1.03–1.14, *p* = 0.0011), SAP (OR = 1.22, 95% CI = 1.15–1.30, *p* = 4.48 × 10^−10^), UAP (OR = 1.26, 95% CI = 1.17–1.35, *p* = 2.49 × 10^−10^), MI (OR = 1.17, 95% CI = 1.08–1.27, *p* = 0.00018), and ischaemic stroke excluding all haemorrhages (OR = 1.0016, 95% CI = 1.0004–1.0027, *p* = 0.0077) (Figure 3B; Supplementary Table S9). Except for CHD, all UVMR results were confirmed through at least one sensitivity analysis. Heterogeneity was not observed among the IVs (Supplementary Table S10). The assessment of the instrument’s validity demonstrated sufficient strength with all F-statistics ≥ 30 (Supplementary Table S5), and no evidence of horizontal pleiotropy was found (all *p* values for egger intercepts ≥ 0.09; Supplementary Table S10). The causal relationships between serum urate concentrations and CHD, SAP, UAP, MI, and ischemic stroke were robustly confirmed using the MRlap approach (Supplementary Table S11), indicating that potential sample overlap had minimal impact on the causal inferences. Similar results were observed for the UVMR analysis performed in the validation datasets for SAP, UAP, and MI (Supplementary Table S9). All sensitivity analyses for CHD supported a significant causal effect of serum urate concentrations. However, non-hemorrhagic stroke was not supported by other sensitivity analyses, and even the weighted median and weighted mode methods yielded opposite directions of effect.

### 3.2. Pleiotropic Analysis of Instrumental Variables

**Figure 4:**
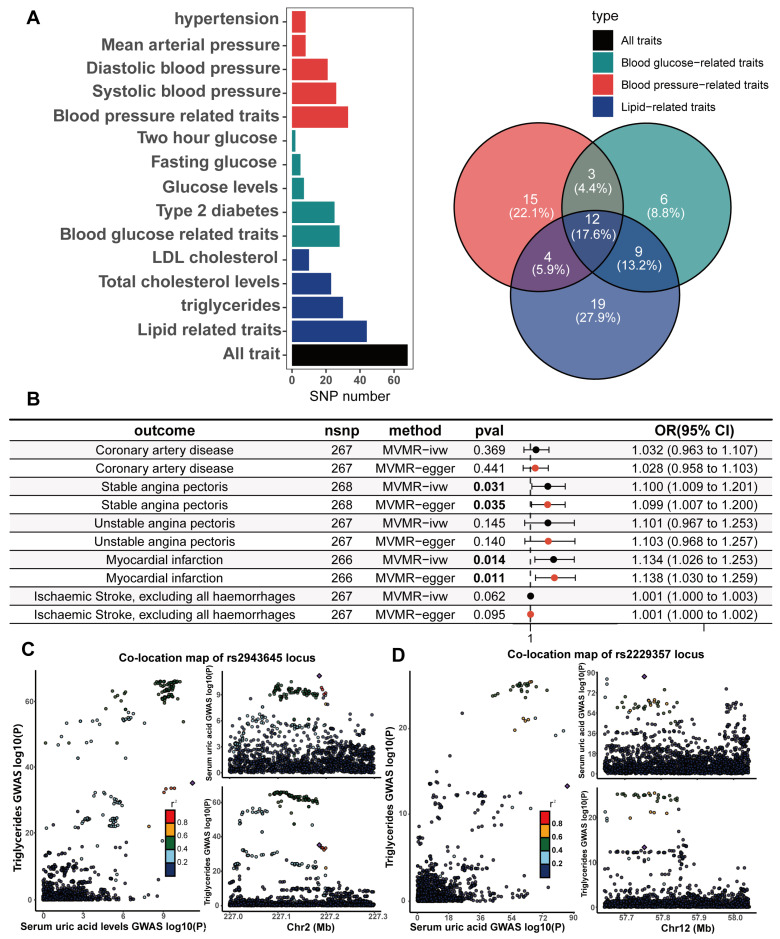
**Pleiotropic examination of genetic instruments; independent causal effects of serum uric acid levels on the occurrence, development, and adverse events of (CHD) were estimated via MVMR**. (**A**) The statistically driven genetic instrument selection strategy yielded a total of 268 SNPs. The association of each SNP with CHD risk factors was examined via LDtrait. The bars show the number of SNPs that are associated with different CHD risk factors. We divided the CHD risk factors into three trait groups, blood pressure-related, glucose-related, and lipid-related traits. The Venn diagram shows the distribution of these SNP associations with different trait groups, with 12 SNPs associated with all three CHD risk factors. (**B**) In the same model, hypertension, diabetes, LDL cholesterol, and serum triglycerides were adjusted, and MVMR was used to assess the independent causal effect of serum uric acid levels on the occurrence of CHD and related adverse events. Results with p values less than 0.05 are bolded. (**C**,**D**) The serum uric acid level and triglyceride level were mapped in the vicinity of rs2943645 (**C**) and rs2229357 (**D**), about 200 kb upstream and downstream. The squares in the figure represent the SNP sites that contribute most to the co-location analysis results.

A potential threat to the reliability of MR studies is a confounding effect due to linkage disequilibrium. This situation arises when a variant linked to the exposure is in linkage disequilibrium with another variant associated with the outcome or confounding variables, leading to a misleading MR association. To examine whether the IVs were associated with known CHD risk factors, we utilized the LDtrait online resource to examine all SNP loci that were in linkage disequilibrium with the IVs. The CHD risk factors we considered included hypertension, diastolic blood pressure, systolic blood pressure, mean arterial pressure, type II diabetes, fasting glucose, random glucose, blood glucose at two hours after the glucose tolerance test, LDL cholesterol, total cholesterol, and triglycerides. The bar and Wayn plots reveal a known pleiotropic profile in these IVs, with 68 of the 268 SNPs being associated with multiple CHD risk factors (Figure 4A; Supplementary Tables S12 and S13). We then performed Bayesian colocalization of serum urate concentrations and CHD risk factors within 20,000 bases upstream and downstream of each instrumental variable to distinguish between horizontal and vertical pleiotropy among them. Eighteen IVs (PPH4 > 0.75) among the sixty-eight SNPs with known pleiotropy were identified that shared the same causal genetic variation with CHD risk factors, which represents a possible vertical pleiotropy (Table 1, Figure 4C,D, Supplementary Table S14).

To adjust for this known pleiotropy, the MVMR analysis was performed to adjust essential hypertension, type 2 diabetes, serum LDL cholesterol, and serum triglycerides together in the same model. In comparison to the primary IVW UVMR analysis, the multivariable MR showed attenuation of the causality between serum urate concentrations and all CHD outcomes. Only the causal associations of serum urate concentrations with SAP (MVMR estimated OR = 1.1, Cl = 1.009–1.2, and *p* = 0.031) and MI (OR = 1.13, Cl = 1.03–1.25, and *p* = 0.014; Figure 4B) remained and were further validated through sensitivity analyses using the MVMR-Egger method (Figure 4B; Supplementary Tables S15 and S16). The causal effects of serum urate concentrations on CHD, UAP, and ischaemic stroke did not persist with the multivariable adjustment. However, the causal effect estimated by MVMR may be underestimated because there is shared causal genetic variation among some of the IVs in which genetics represent serum urate and diabetes, hypertension, triglycerides (Figure 4C,D), and LDL cholesterol.

It should be noted that MVMR cannot account for unmeasured pleiotropic effects. We used MR-Egger and MR-PRESSO to test whether unknown pleiotropy affects the causal estimates and corrected for the effects of unknown pleiotropy. The MR-Egger intercept analysis did not detect any indication of asymmetric horizontal pleiotropy between serum urate concentrations and CHD (egger_intercept *p* = 0.094), SAP (egger_intercept *p* = 0.33), UAP (egger_intercept *p* = 0.28), MI (egger_intercept *p* = 0.98), and ischaemic stroke (egger_intercept *p* = 0.276) (Supplementary Table S10). The *p*-values obtained from the global test in the MR-PRESSO analysis suggest a potential for pleiotropic effects between serum urate concentrations and CHD, SAP, UAP, and MI (Supplementary Table S17). However, after excluding the outliers, the MR-PRESSO outcomes continued to indicate a statistically significant causality between serum urate concentrations and CHD (*p* = 3.91 × 10^−6^), SAP (*p* = 1.57 × 10^−8^), UAP (*p* = 0.0031), and MI (*p* = 4.21 × 10^−7^) (Supplementary Table S18).

### 3.3. Causality Between Serum Urate Concentrations and CHD Risk Factors Based on the Biologically Driven Genetic Instrument Selection Strategy

Considering the shared genetic variation between serum urate concentrations and hypertension, systolic blood pressure, type II diabetes, LDL cholesterol, and serum triglycerides, adjustment for these CHD risk factors in MVMR models may lead to an underestimation of causal relationships. We further explored the causality between serum urate concentrations and the CHD, SAP, and MI and risk factors of CHD via a biological mechanism-driven genetic instrument selection strategy. A total of 89 SNPs were obtained (Supplementary Table S6). Pleiotropic examination by LDtrait did not reveal an association between these genetic tools and the CHD risk factors (Supplementary Table S19). The FORGEdb scores for each genetic instrument varied from 2 to 10. The linkage disequilibrium statistic (r2) for each genetic instrument was less than 0.35 (Supplementary Figure S1A–E; Supplementary Table S8). Our primary analysis of the inverse variance weighting method revealed significant causality between the serum urate concentrations and CHD (OR = 1.047, 95% CI = 1.019–1.076, *p* = 0.00083), SAP (OR = 1.052, 95% CI = 1.003–1.103, *p* = 0.024), and MI (OR = 1.106, 95% CI = 1.057–1.157, *p* = 1.41 × 10^−5^). The UVMR results of CHD and MI were confirmed by at least one additional sensitivity analysis. Although the UVMR results for stable angina failed to gain support from other sensitivity analyses, the causality between the serum urate concentrations and SAP was verified by the MRlap and MR-PRESSO methods. Therefore, we still believe that a causal link exists between them. In the significant causality, heterogeneity was not observed among the IVs (Supplementary Table S21). The assessment of instrumental validity indicated the adequate strength of the instruments (all F-statistics ≥ 30; Supplementary Table S6). No evidence of horizontal pleiotropic effects was found in the causality between the serum urate concentrations and CHD, SAP, and MI (Supplementary Table S21). The causal relationship remained significant following the exclusion of outliers using the MR-PRESSO method (Supplementary Table S23). The causal associations between the serum urate concentrations and CHD, SAP, and MI were robustly confirmed through the application of the MRlap method (Supplementary Table S22), implying that potential sample overlap may not significantly influence the causal conclusions. Similar results were observed for the UVMR analysis performed in the validation datasets for SAP, UAP, and MI (Supplementary Table S20).

### 3.4. Mediators Between the Serum Urate Concentrations and CHD, SAP and MI

**Figure 5:**
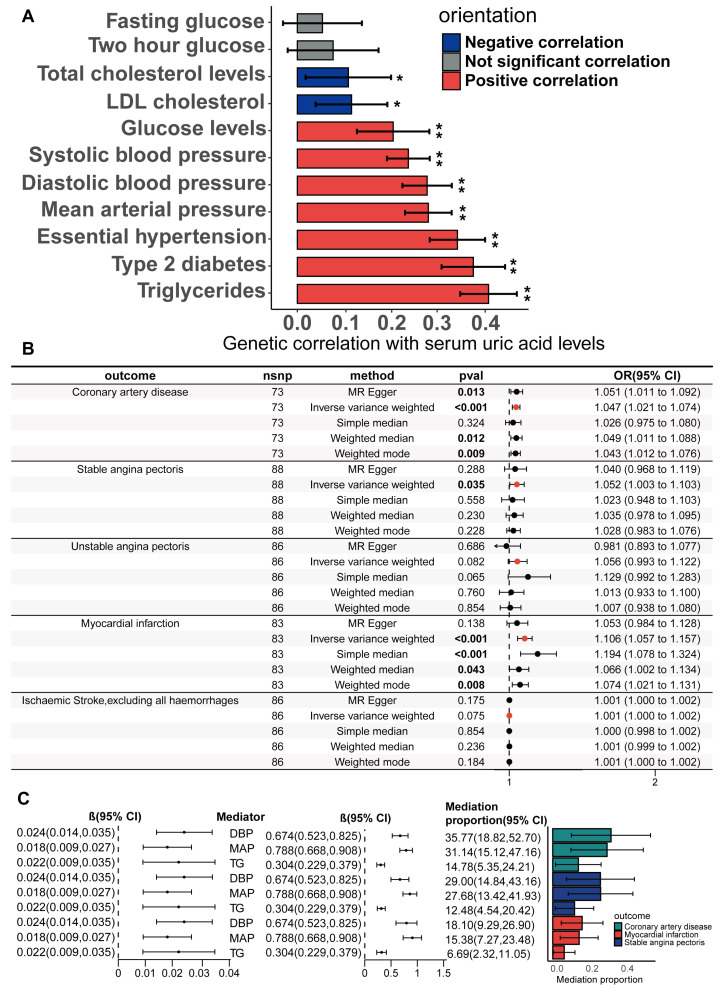
**Genetic correlation of serum uric acid levels with CHD risk factors; estimation of the mediating effect of CHD risk factors**. (**A**) The genetic correlation of serum uric acid levels with risk factors for CHD was estimated via linkage disequilibrium regression analysis. Values are shown as rg ± SE. * indicates that the hypothesis test result is less than the *p*-value calculated via the Benjamin–Hochberg method, ** indicates that the hypothesis test result is less than the *p*-value of significance calculated via the Bonferroni method. (**B**) Univariate two-sample Mendelian randomization methods to estimate the causal effect of serum uric acid levels on coronary heart disease and related adverse events using a biologically driven genetic tool selection strategy. (**C**) Two-step mediation Mendelian randomization to estimate the mediation effect of diastolic blood pressure, mean arterial pressure, and serum triglycerides. The left forest plot shows the causal effect of serum uric acid levels on diastolic blood pressure, mean arterial pressure, and serum triglycerides. The right forest plot shows the causal effects of diastolic pressure, mean arterial pressure, and serum triglycerides on CAD, stable angina, and myocardial infarction. The LDSC data are presented as genetic correlation (rg) ± SE. The Mediation proportion are presented as estimated value ± SE.

We further investigated the potential mediating effects of CHD risk factors in the causal relationship between the serum urate concentrations and CHD, SAP, and MI, using IVs obtained through biologically driven genetic tool selection strategies. We selected hypertension, systolic blood pressure, mean arterial pressure, diastolic blood pressure, type II diabetes, fasting blood glucose, random blood glucose, blood glucose two hours after the glucose tolerance test, total cholesterol, LDL cholesterol, and serum triglycerides as candidate mediator factors. First, the genetic correlation of serum urate concentrations with these candidate mediating factors was calculated via LDSC regression analysis. Through the application of cross-trait LDSC analysis, statistically significant genetic correlations were observed between serum urate concentrations and essential hypertension, diastolic blood pressure, systolic blood pressure, mean arterial pressure, type 2 diabetes, random blood glucose, total cholesterol levels, LDL cholesterol, and triglycerides in the European population (range of absolute values of rg, |0.11| to |0.39|; *p*  =  4.43 × 10^−40^ to 0.0195; Figure 5A; Supplementary Table S4). For CHD risk factors that have a significant genetic correlation with serum urate concentrations, we subsequently performed the UVMR analyses to estimate the causality. Our primary analysis of IVW unveiled significant causality between serum urate concentrations and diastolic blood pressure (beta = 0.025, 95%CI = 0.015–0.034, and *p* = 1.3 × 10^−7^), mean arterial pressure (beta = 0.018, 95%CI = 0.009–0.027, and *p* = 0.0019), and triglycerides (beta = 0.018, 95%CI = 0.009 −0.028, and *p* = 0.00019) (Supplementary Figure S2; Supplementary Tables S20 and S24). For the significant causal associations, all UVMR outcomes underwent validation through at least one sensitivity analysis. No statistically significant evidence of horizontal pleiotropy was identified in our analysis (all *p* value for egger intercepts ≥ 0.35; Supplementary Table S21). Following the exclusion of outliers, the results obtained from the MR-PRESSO analysis continued to indicate a statistically significant association between serum urate concentrations and triglycerides (*p* = 0.0009), mean arterial pressure (*p* = 0.0002), and diastolic blood pressure (*p* = 5 × 10^−6^) (Supplementary Table S23). The causal associations were robustly confirmed through the MRlap method (Supplementary Table S22), implying that potential sample overlap may not significantly influence the causal conclusions. We also verified the causality of diastolic blood pressure, mean arterial pressure, and triglycerides with CHD, SAP, and MI via UVMR(Supplementary Figure S3). Preliminary analyses of the IVW data and all sensitivity analyses supported the presence of significant causal relationships (Supplementary Table S25). The above results suggest that diastolic pressure, mean arterial pressure, and serum triglycerides may mediate the causal relationships between serum urate concentrations and CHD, SAP, and MI. We estimated the mediation effect and effect proportion of these mediators via the two-step MR. Among the total effects of serum urate concentrations on CHD, SAP, and MI, the mediation effect of diastolic blood pressure was the greatest, reaching 36.7%, 41.0%, and 19.4%, respectively (Figure 5C; Supplementary Tables S26 and S27).

### 3.5. Potential Mechanisms Mediating the Causality Between Serum Urate and CHD

**Figure 6:**
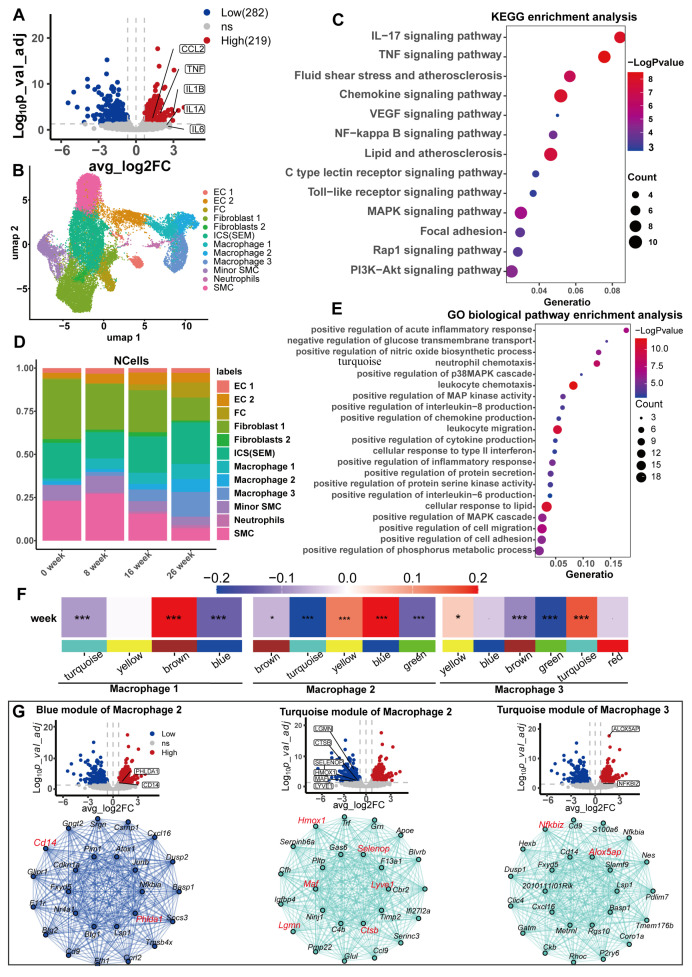
Transcriptome analysis of human monocytes exposed to uric acid in vitro and single-cell transcriptome analysis of arterial tissue in a mouse model of atherosclerosis. (**A**) Volcano plot of uric acid-induced differential genes in human monocytes, with tags labeling signature proinflammatory markers of M1 macrophages. (**B**) UAMP visualization of single-cell transcriptome data, with a representative cell type for each cluster for all combined time points (0, 8, 16, and 26 weeks). Vascular endothelial cell (EC), fibroblast (FC), smooth muscle cell (SMC), intermediate cell state (ICS), and SMC-derived intermediate cells (SEM). (**C**,**E**) The results of differential gene enrichment analysis of upregulated genes in isolated human monocytes induced by uric acid. KEGG enrichment analysis (**C**); GO biological pathway enrichment analysis (**E**). (**D**) Proportion of cell types at different time points. (**F**) Heatmap of correlations between weighted coexpression gene modules and molding time for the three macrophage subtypes. The colored narrow bands were used to distinguish between the different gene co-expression modules, and the colored wide bands indicate the correlation between the different gene modules and the atherosclerotic lesions. * *p* < 0.05; and *** *p* < 0.001. (**G**) Volcano plots of human monocytes exposed to uric acid in vitro and Hub gene network map of key gene modules. From left to right are the blue module of macrophage 2, the turquoise module of macrophage 2, and the turquoise module of macrophage 3.

Atherosclerosis arises due to the retention of apolipoprotein B-containing lipoproteins within the subendothelial space, initiating a chronic, unresolved inflammatory cascade that progressively fuels the advancement of the disease over time [49,50,51]. Macrophages are the predominant immune cells within atherosclerotic lesions, originating primarily from myeloid progenitors in the bone marrow [52]. The persistent presence of monocytes exhibiting low-grade inflammatory characteristics contributes to the progress of atherosclerosis [53]. The harmful effects of high concentrations of serum urate on CHD found in observational studies are mainly caused by excessive inflammation and elevated blood pressure [54]. In vitro, urate stimulation induced a macrophage transition to a proinflammatory phenotype [55,56]. We hypothesized that high concentrations of serum urate may induce and maintain the proinflammatory phenotype of macrophages in atherosclerotic lesions. We used the GSE107821 transcriptome data downloaded from the GEO database (Supplementary Figure S4A,B). We first performed differential analysis (Supplementary Table S28) and differential gene enrichment analysis on the transcriptomes of monocytes exposed to urate in vitro. After 24 h, monocytes exposed to urate highly expressed proinflammatory cytokines, including interleukin 6, interleukin 1β, tumor necrosis factor (TNF), and the monocyte chemokine CCL2 (Figure 6A). Our enrichment analysis of upregulated DEGs suggested that the biological functions of these genes were mainly with proinflammatory signaling pathways and atherosclerosis (Figure 6C,E).

We next analyzed whether similar transcriptomic changes exist in macrophages from an atherosclerotic mouse model (Ldlr-/-mice) and whether these transcriptomic changes were important for the progression of atherosclerosis. Eight arterial tissue samples from Ldlr-/- mice fed a Western diet were downloaded from the GSE155513 dataset, corresponding to time points of 0, 8, 16, and 26 weeks, respectively. The scRNA-seq data underwent extensive quality control measures and preprocessing procedures to ensure robustness and reliability (Supplementary Figure S4D and Figure 6A–E). We observed the high data quality of the samples, high unique molecular identifier (UMI) and gene counts per cell, and low proportions of mitochondrial RNA and ribosomal RNA. We ultimately obtained a total of 27,282 post-QC cells (Supplementary Figure S4C). Cluster analysis identified 12 cell clusters (Figure 6B). We identified the marker genes for each cell cluster, annotated the cell clusters based on the marker genes of the original study and the PanglaoDB database [47], and drew a bubble diagram to visualize the relationships (Supplementary Figure S4E). There were three macrophage subsets. With the progression of atherosclerotic lesions, the proportion of all three types of macrophages obviously increased (Figure 6D). We used hdWGCNA to identify key molecular features associated with atherosclerosis progression in macrophages. Four, five, and six gene modules were identified in each of the three macrophage subtypes (Supplementary Figure S6A,B). The connectivity of the genes in each gene module was calculated, and the top 20 connected genes, namely, the Hub gene, were treated as key genes in the module. The correlation of each gene module with atherosclerotic plaque progression was calculated to identify gene modules significantly associated with lesion progression. The brown module of macrophage 1, the blue module of macrophage 2, and the turquoise module of macrophage 3 were significantly and positively correlated with atherosclerotic plaque progression. The turquoise module of macrophage 2 and the green module of macrophage 3 were significantly negatively correlated with lesion progression (Figure 6F; Supplementary Table S31). We performed KEGG enrichment analysis for genes constituting the five modules separately, where the blue module of macrophage 2, the turquoise module, and the turquoise module of macrophage 3 were enriched in atherosclerosis (Supplementary Figure S6C–F). The green module of macrophage 3 was not enriched in any meaningful pathways or diseases. We considered these three gene modules as key modules in the progression of atherosclerosis, with the Hub gene as the key gene (Supplementary Table S30). We compared these Hub genes with the differentially expressed genes from human monocytes exposed to urate in vitro and identified 10 genes (Figure 6G). Interestingly, all the upregulated DEGs were significantly positively correlated with atherosclerotic lesion progression, whereas the downregulated DEGs were significantly negatively correlated with atherosclerotic lesion progression. These findings suggest that the effect of urate on the macrophage transcriptome principally promotes the progression of atherosclerosis. Additionally, these 10 genes are potential mediators. We conducted an enrichment analysis on these 10 genes. The four upregulated genes were exclusively enriched in the inflammatory response pathway (Supplementary Figure S7A online), while the six downregulated genes predominantly clustered in the leukocyte migration pathway (Supplementary Figure S7B). These findings demonstrate that serum uric acid levels can induce macrophage inflammation and impair their migratory capacity, thereby trapping macrophages within atherosclerotic lesions and accelerating the pathological progression of atherosclerosis.

## 4. Discussion

In this study, we found a significant genetic correlation between serum urate concentrations and CHD, MI, SAP, hypertension, type II diabetes, and serum triglyceride levels via the cross-trait LDSC analysis. This finding does not represent a causal relationship, but highlights the complexity of the relationship between serum urate concentrations and the occurrence, development, and related adverse events of CHD. We obtained a total of 268 SNPs via a statistically driven genetic tool selection strategy, and after pleiotropic examination, we identified 68 SNPs associated with CHD risk factors, which implies pleiotropic effects of those genetic instruments. We distinguished between horizontal and vertical pleiotropy by Bayesian colocalization analysis and found that a total of 18 SNP loci were common causal genetic variants between serum urate concentrations and risk factors of CHD. These risk factors include hypertension, diastolic blood pressure, systolic blood pressure, type II diabetes mellitus, and serum triglyceride levels. This means that there may be a causality between the serum urate concentrations and these CHD risk factors. The results of MR indicate that there is a causality between serum urate concentrations and SAP and MI independent of CHD risk factors. This result was replicated in MR via biologically driven genetic tool selection strategies. Furthermore, a causal effect of serum urate concentrations on mean arterial pressure, diastolic blood pressure, and serum triglycerides was found. Mediation analysis revealed that mean arterial pressure, diastolic blood pressure, and serum triglycerides partially mediated the causal effects of serum urate concentrations on CHD, SAP, and MI. However, the bias in the mediation analysis will increase because of the interaction between serum urate concentrations and diastolic blood pressure, mean arterial pressure, and serum triglycerides.

Our research possessed notable merits. Our MR analysis incorporated more powerful instruments for serum urate concentrations than were available previously. Genetic tools come from data from the largest studies of population size and SNP testing. The genetic variants employed as IVs in our study accounted for approximately 13.3 percent of the variability observed in serum urate concentrations. We used two genetic instrument selection strategies to provide consistent evidence of the causal effect of serum urate concentrations on CHD, SAP, and MI. We performed a comprehensive examination of the pleiotropy of the genetic instruments. LDSC regression analysis and Bayesian colocalization analysis provided insight into the complex relationship between serum urate concentrations and CHD risk factors. We performed a comprehensive assessment of the robustness of the MR results via multiple methods, including MR-PRESSO analysis, MR-Egger analysis, MRlap analysis, etc.

There are also some limitations in our research. Limitations include most of the data from genome-wide association studies by Saori Sakaue et al. [33], which resulted in overlap between the two samples used for MR analysis. Nevertheless, we corrected for the possible impact of sample overlap on the results via the MRlap method. In addition, we replicated our finding using the validation datasets. Owing to the difficulty in searching a validation cohort for UAP completely independent from the UK Biobank in the European population, we did not validate our result of UAP in an independent cohort. However, we make up for this deficiency via MR on the basis of two different genetic instrument selection strategies. Furthermore, the bidirectional relationship between serum urate concentrations and both diastolic blood pressure and serum triglycerides would increase the error in the estimation of mediation effects proportion. Finally, our study is mainly based on individuals of European descent and may not be applicable to other populations or settings.

An observational study revealed a positive correlation between oxLDL-cholesterol levels and serum urate concentrations [57]. However, in our study, LDSC regression analysis, Bayesian colocalization, and MR analysis all indicated that there was no causality between the serum urate concentrations and LDL-cholesterol. The positive association between serum urate concentrations and type II diabetes reported in observational studies was not reliably reproduced in our study. However, significant genetic correlation, shared causal genetic variation, and positive results of the MR-Egger method in MR analysis suggest a potential causal relationship between them. In addition, our first stage of Mendelian randomization also found a potential causal relationship between serum uric acid levels and ischemic stroke. Although this result was not replicated in our second phase study, other MR studies found a significant causal relationship between serum uric acid levels and venous thromboembolic disease [58].

The causal effect of serum urate concentrations on SAP and MI that we found is reasonable and meaningful. Endothelial dysfunction is an important cause of atherosclerotic plaque formation. Human endothelial cells express urate transporters, and hyperuricaemia can cause endothelial cell dysfunction through a variety of mechanisms [59,60,61]. The causal effect of serum urate concentrations on diastolic pressure and triglycerides has also been supported by other studies. A study of the temporal relationship between hyperuricaemia and hypertension revealed that increased serum urate concentrations may precede increased blood pressure, and that blood pressure partly mediates the effects of serum urate concentrations on new cardiovascular disease [23]. Furthermore, excessive inflammation may also play an important mediating role in the effect of elevated urate concentrations on cardiovascular disease risk [54]. A direct causal effect of hyperuricaemia on hypertension has also been found in a rat model of hyperuricaemia [62]. Prior research likewise indicated that the urate-lowering drugs allopurinol and febutostat can reduce triglyceride levels in fructose-fed rats [63,64,65]. Urate promoted triglyceride synthesis in mouse primary hepatocytes through the overexpression of the fatty acid synthase (FAS)stearoyl-CoA desaturase 1 (SCD1) and lipogenic enzyme acetyl-CoA carboxylase 1 (ACC1) via the activation of SREBP-1c [66].

Our transcriptomic analysis revealed that urate-induced macrophage transcriptomic changes are associated with the progression of atherosclerotic lesions and may play an important role in promoting lesion development. We first analyzed the uric acid treated in vitro macrophage RNAseq dataset to identify the differential genes. Then we analyzed single-cell transcriptome data from an atherosclerotic plaque sample and found the macrophages. We identified the gene set in macrophages that is significantly associated with the progression of atherosclerotic lesions. Then, the intersection of the aforementioned differential genes and the genes associated with the progression of atherosclerotic lesions was selected. A total of 10 overlapping genes were identified, among which 4 genes were induced up and 6 genes were down by uric acid. Interestingly, the four upregulated genes were only found in the gene cluster with significantly positive correlation with the atherosclerotic disease process, while the six downregulated genes were only found in the gene cluster with significantly negative correlation with the disease process. This suggests that the effect of uric acid on the transcriptome of macrophages is completely promoting atherosclerosis progression. GO enrichment analysis revealed that uric acid promoted the inflammatory response of macrophages, weakened the migration ability of macrophages, and promoted their retention in pathological sites, ultimately promoting the progression of atherosclerotic lesions. However, our study was limited to the analysis of transcriptome data, and the above findings were not verified by experiments.

Ten key genes possibly mediating the promoting effect of urate on atherosclerotic lesions were identified. The relationships of these 10 key genes with atherosclerosis have been reported in previous studies, although some studies have been limited to observational studies or genetic studies. Among them, genetic variation at the rs3217713 locus in the NFKBIZ gene emerges as a standalone predictor for the development of early-stage CHD [67]. Genetic variation in the ALOX5AP gene is also considered to be associated with a differential risk of CAD [68]. 5-lipoxygenase (ALOX5AP) is abundantly expressed in the arterial wall of patients with different lesion stages of atherosclerosis [69]. Heme oxygenase (HMOX1) plays an important role in the development and homeostatic maintenance of the vascular system, and human HMOX1-deficient patients exhibit systemic inflammation and vascular damage. Compared with humans, HMOX1^-/-^ mice exhibit a similar phenotype, particularly vascular damage and signs of increased adhesion of monocytes to the vascular wall [70]. The SELENOP gene encodes a selenoprotein that contains multiple selenocysteine (Sec) residues per peptide (10 in humans), accounts for the majority of selenium in plasma and is considered an extracellular antioxidant. The available evidence on the protective effect of selenoproteins on cardiovascular diseases, such as atherosclerosis, was summarized by Diane E Handy and Hongmei Liu et al. [71,72]. The protein encoded by the MAF gene is a DNA-bound, leucine zipper-containing transcription factor identified as a novel central regulator of the atherosclerosis/CHD-associated liver network and may be a potential therapeutic target [73]. Recent studies published in nature found that macrophages expressing LYVE1 are atheroprotective [74]. HMOX1, SELENOP, and LYVE1 are the Hub genes identified in our study that have significant negative associations with the development of coronary atherosclerotic lesions. However, the Lgmn and Ctsd reported in our study are not consistent with the existing findings.

## 5. Conclusions

In conclusion, this MR study elucidated the independent causal effect of serum urate on SAP and MI, where serum triglycerides and diastolic blood pressure partially mediated this effect. Transcriptomics analysis revealed that serum urate may have a direct effect on atherosclerosis by inducing transcriptomic changes in macrophages. Our findings highlight that the control of serum urate concentration in the long-term management of CHD patients may be necessary. Well-designed clinical trials and foundational research are presently required to furnish conclusive proof regarding the specific clinical scenarios in which adequate reduction in urate concentrations can confer cardiovascular advantages.

## Figures and Tables

**Table 1:** The SNPs with vertical pleiotropy identified by colocalization analysis.

SNP_UA	Position (GRCh37)	GWAS Trait	PMID	SNP_Other_Trait	Position (GRCh37)	r^2^	*p*-Value	PP.H3.abf	PP.H4.abf
rs77924615	16:20392332	Diastolic blood pressure	38689001	rs77924615	chr16:20392332	1	2.00 × 10^−39^	1.06 × 10^−6^	0.99999893
rs72681698	14:51207741	Diastolic blood pressure	35762941	rs72677850	chr14:50849397	1	3.00 × 10^−10^	1.78 × 10^−5^	0.99998222
rs2219647	16:51733405	Diastolic blood pressure	38689001	rs9932220	chr16:51758116	0.89426289	2.00 × 10^−13^	0.22711367	0.77288099
rs2219647	16:51733405	Essential hypertension	32589924	rs9932220	chr16:51758116	0.89426289	2.00 × 10^−8^	0.00491713	0.99464745
rs963837	11:30749090	Systolic blood pressure	33230300	rs3925584	chr11:30760335	0.955974843	3.00 × 10^−9^	0.11073903	0.88785727
rs77924615	16:20392332	Systolic blood pressure	38689001	rs77924615	chr16:20392332	1	2.00 × 10^−21^	1.94 × 10^−6^	0.99999805
rs72681698	14:51207741	Systolic blood pressure	30578418	rs72677850	chr14:50849397	1	2.00 × 10^−16^	0.18104193	0.81895644
rs2219647	16:51733405	Systolic blood pressure	38689001	rs9932220	chr16:51758116	0.89426289	1.00 × 10^−10^	0.23499299	0.76500700
rs2823139	21:16576783	Systolic blood pressure	30578418	rs2823139	chr21:16576783	1	9.00 × 10^−12^	0.22394859	0.77605130
rs11128603	3:12385828	Triglyceride levels	34887591	rs1801282	chr3:12393125	1	5.00 × 10^−26^	0.00035804	0.99964193
rs2060824	2:61484556	Triglyceride levels	34887591	rs766448	chr2:61735446	0.915200932	4.00 × 10^−11^	1.49 × 10^−8^	0.99999998
rs146787580	2:203412513	Triglyceride levels	32203549	rs3731696	chr2:203431804	0.916666667	6.00 × 10^−13^	5.21 × 10^−9^	0.99999999
rs2012385	2:242422405	Triglyceride levels	32203549	rs4675812	chr2:242395674	0.711111111	1.00 × 10^−12^	4.32 × 10^−9^	0.99999999
rs2943645	2:227099180	Triglycerides	34594039	rs56256300	chr2:227098186	1	7.00 × 10^−67^	4.66 × 10^−9^	0.99999999
rs1260326	2:27730940	Triglycerides	34594039	rs1260326	chr2:27730940	1	7.00 × 10^−102^	3.50 × 10^−8^	0.99999996
rs114165349	1:27021913	Triglycerides	34594039	rs114165349	chr1:27021913	1	4.00 × 10^−29^	9.05 × 10^−8^	0.99999991
rs2229357	12:57843711	Triglycerides	34594039	rs2122982	chr12:57781893	1	3.00 × 10^−26^	0.00026704	0.99973295
rs10211562	2:111930796	Triglycerides	34594039	rs10211562	chr2:111930796	1	1.00 × 10^−10^	9.34 × 10^−9^	0.99999999
rs12096443	1:50984962	Triglycerides	30275531	rs1278530	chr1:50889255	0.854051968	8.00 × 10^−12^	1.60 × 10^−6^	0.99999839
rs1260326	2:27730940	Two-hour glucose	34059833	rs1260326	chr2:27730940	1	6.00 × 10^−12^	6.31 × 10^−5^	0.99958036
rs1260326	2:27730940	Type 2 diabetes	34594039	rs1260326	chr2:27730940	1	6.00 × 10^−29^	3.71 × 10^−13^	1
rs11128603	3:12385828	Type 2 diabetes	30595370	rs17036160	chr3:12329783	1	1.00 × 10^−11^	0.00098573	0.99901423
rs76895963	12:4384844	Type 2 diabetes	30595370	rs76895963	chr12:4384844	1	2.00 × 10^−31^	4.38 × 10^−7^	0.99999956
rs62106258	2:417167	Type 2 diabetes	30297969	rs62107261	chr2:422144	1	4.00 × 10^−12^	0.01668743	0.92439756
rs10899125	11:75517332	Type 2 diabetes	32541925	rs11236524	chr11:75464344	1	1.00 × 10^−8^	5.45 × 10^−7^	0.99999945
rs10211562	2:111930796	Type 2 diabetes	30054458	rs10169613	chr2:111934977	0.936140351	4.00 × 10^−8^	9.85 × 10^−7^	0.99999901

A SNP with a PP.H4.abf > 0.75 was considered to be a causal genetic variant shared by both serum uric acid levels and CHD risk factors.

## Data Availability

Summary GWAS data used for the Mendelian randomization analysis were extracted from the IEU open GWAS project (https://gwas.mrcieu.ac.uk/) (21 July, 2024), GWAS catlog (https://www.ebi.ac.uk/gwas/) (10 July, 2024) and dbGap database (https://www.ncbi.nlm.nih.gov/gap/advanced_search/?OBJ=analysis&COND=%7B”study_accession”:%5B”phs002453.v1.p1”%5D%7D) (13 March, 2025). The unique identifiers for each trait can be found in Supplementary Table S2. Complete summary GWAS data used for Bayesian colocalization and LDSC analysis were dowloaded from the IEU open GWAS project and GWAS catalog. Bulk-RNAseq data and single-cell sequencing data were obtained from the Gene Expression Omnibus (GEO) of the National Center for Biotechnology Information. They can be accessed through the GEO serial accession numbers GSE107821 and GSE155513. The results of this study can be obtained by contacting the corresponding author. The datasets supporting the conclusions of this article are included within the article and its additional file. The other data generated or analyzed during this study are available in this published article and its Supplementary Information files.

## Supplementary Materials

The following supporting information can be downloaded at: https://www.mdpi.com/article/10.3390/biomedicines13081838/s1. Figure S1: Heatmap of the linkage disequilibrium coefficient of SNPs; Figure S2: Estimation of the causal relationship between the serum uric acid level and CHD risk factors (biologically driven genetic tool selection strategy); Figure S3: Direct effects of CHD risk factors on the occurrence, development and adverse events of CHD; Figure S4: Bulk RNA-seq and single-cell RNA-seq analyses; Figure S5: Overview of the quality control of the scRNA-seq data.; Figure S6: Weighted gene co-expression network analysis for the three macrophage cell types; Figure S7: Enrichment analysis of 10 hub genes. Table S1: STROBE Statement-Checklist of items in reports of Mendelian randomization; Table S2: Overview of the GWAS data used in the study; Table S3: Description of the genes involved in urate metabolism and transport; Table S4: Genetic correlation estimates from LDSC regression; Table S5: Overview of the SNPs from statistically driven genetic instrument selection strategy; Table S6: Overview of the SNPs from biologically driven genetic instrument selection strategy; Table S7: SNPs removed from heterogeneity testing and data harmonization; Table S8: Matrix of linkage disequilibrium relationships of the SNPs from biologically driven genetic instrument selection strategy; Table S9: UVMR estimates for the causal associations between UA and CHD (statistically driven genetic instrument selection strategy); Table S10: Directional pleiotropy test and heterogeneity test for the causal associations between UA and CHD (statistically driven genetic instrument selection strategy); Table S11: MRlap estimates for the causal associations (statistically driven genetic instrument selection strategy); Table S12: The pleiotropic examination of the SNPs from statistically driven genetic instrument selection strategy; Table S13: Summary of the pleiotropy of SNPs from statistically driven genetic instrument selection strategy; Table S14: The Bayesian colocalization results of these promiscuous pleiotropic SNPs; Table S15: MVMR estimates for the causal associations between UA and CHD with adjustment for CHD risk factors in the same model.(statistically driven genetic instrument selection strategy); Table S16: Directional pleiotropy test for the causal associations between UA and CHD in MVMR. (statistically driven genetic instrument selection strategy); Table S17: The MR-PRESSO global test of the causal associations between UA and CHD.(statistically driven genetic instrument selection strategy); Table S18: The MR-PRESSO analysis results of the causal associations between UA and CHD.(statistically driven genetic instrument selection strategy); Table S19: The pleiotropic examination of the SNPs from biologically driven genetic instrument selection strategy; Table S20: UVMR estimates for the causal associations between UA and CHD, and CHD risk factors. (biologically driven genetic instrument selection strategy); Table S21: Directional pleiotropy test and heterogeneity test for the causal associations between UA and CHD, and CHD risk factors (biologically driven genetic instrument selection strategy); Table S22: MRlap estimates for the causal associations between UA and CHD, and CHD risk factors. (biologically driven genetic instrument selection strategy); Table S23: The MR-PRESSO analysis results of the causal associations between UA and CHD, and CHD risk factors.(biologically driven genetic instrument selection strategy); Table S24: UVMR estimates for the causal effect of each mediator on serum uric acid levels.; Table S25: UVMR estimates for the causal association between each mediator and CHD, SAP, and MI.; Table S26: MVMR estimates for the causal association between each mediator and CHD, SAP, and MI with adjustment for UA.; Table S27: Mediation MR analysis results.; Table S28: Transcriptome differential transcriptiome genes in human monocytes exposed to uric acid in vitro; Table S29: Single-cell transcriptome QC parameter table; Table S30: List of gene connectivity in the three types of macrophage gene modules; Table S31: Correlations of gene modules of macrophages with progression of atherosclerotic lesions. References [75,76,77,78,79,80,81,82,83,84,85] are cited in Supplementary Materials.

## Abbreviations

The following abbreviations are used in this manuscript:

CHD	coronary heart disease
SAP	unstable angina pectoris
MI	myocardial infarction
SNPs	single nucleotide polymorphisms
IVs	instrumental variables
MR	Mendelian randomization
MR-PRESSO	Mendelian randomization pleiotropy RESidual sum and outlier
MRlap	Mendelian randomization with Lasso penalty
GWAS	genome-wide association study
IVW	inverse variance weighted
OR	odds ratios
LD	linkage disequilibrium
LDL	low density lipoprotein
PC	principal components
PPH3	posterior probability for hypothesis 3
PPH4	posterior probability for hypothesis 4
KEGG	Kyoto Encyclopedia of Genes and Genomes
GO	gene ontology
scRNA	single-cell RNA sequencing
DEGs	differentially expressed genes
TNF	tumor necrosis facto
CCL2	C-C motif chemokine ligand 2
CD14	cluster of differentiation 14
PHLDA1	pleckstrin homology like domain family A member 1
HMOX-1	heme oxygenase 1
LYVE1	lymphatic vessel endothelial hyaluronan receptor 1
SELENOP	selenoprotein P
MAF	MAF bZIP transcription factor
CTSB	cathepsin B
LGMN	legumain
NFKBIZ	nuclear factor kappa B inhibitor zeta
ALOX5AP	arachidonate 5-lipoxygenase activating protein
CARDIoGRAMplusC4D	plus C4D The Coronary ARtery DIsease Genome wide Replication and Meta-analysis plus The Coronary Artery Disease Genetics
WD	Western diet
EC	vascular endothelial cell
FC	fibroblast
SMC	smooth muscle cell
ICS	intermediate cell state
SEM	SMC-derived intermediate cells
