# Supplementary material for: Causal Relationship Between Serum Uric Acid and Atherosclerotic Disease: A Mendelian Randomization and Transcriptomic Analysis
Source: Biomedicines. 2025 Jul 28;13(8):1838. doi: 10.3390/biomedicines13081838 (PMC12383975; doi:10.3390/biomedicines13081838)
Supplement: Supplementary file 1 [file biomedicines-13-01838-s001.zip › biomedicines-3759179-supplementary/Supplemental material.pdf]

Supplementary Method

1.UVMR and MVMR analysis

MR analysis is a form of instrumental variable analysis that employs genetic variants as proxy instrumental variables (IVs) to investigate causality. MR analysis rests on three core assumptions: (1) the genetic instruments are associated with the exposure variable; (2) the genetic instruments are unconfounded by any extraneous factors; (3) the genetic instruments exert their influence on outcomes solely through the exposure.

Coordination of the exposure data and outcome data is required during performing a UVMR analysis for each exposure outcome combination. This implies that for a SNP, its effect on the exposure must align with its effect on the outcome, both pertaining to the same allele. We deduce the alleles of the forward strand by utilizing allele frequency data. We discarded the SNP which failed to infer the forward strand alleles. For each UVMR analysis of the combination of exposure outcomes, we removed the genetic instruments that brought greater heterogeneity. The specific method is as follows: We measured the influence of the genetic instruments on the heterogeneity by testing the remaining genetic tools for the heterogeneity after excluding each SNP. Then, following this prioritization, the SNP was sequentially removed until a robust UVMR result with no significant heterogeneity was obtained. Supplementary table 7 describes the SNP and rationtification for elimination in the UVMR analysis for each combination of exposure outcome.

Provided that the IVs adhere to the assumptions of MR, the inverse variance weighted (IVW) method yields the highly accurate, unbiased, and efficient estimations of causal effects.. Furthermore, the random-effects IVW method is robust for potential heterogeneity among the IVs[1]. For our UVMR analysis, we primarily employed the random-effects inverse variance-weighted approach as the core methodological tool[2],and sensitivity analyses, encompassing the weighted median estimator (WM), MR-Egger regression, the simple median approach, the weighted mode estimator, and the MR-PRESSO test, were conducted to further assess the robustness of the results. Furthermore, we employed the MRlap analysis to confirm the causal links, where there was an overlap of samples between the exposure and outcome datasets. The MRlap approach is capable of addressing biases in causal effect estimates that arise due to overlapping samples[3]. Moreover, we computed the intercepts from the MR-Egger regression and the MR-PRESSO global test to assess the presence of potential unaccounted-for pleiotropic effects. In cases where the p-values from the global test in the MR-PRESSO analysis were below 0.05, the MR-PRESSO estimates reported were those obtained after the removal of outliers.

MVMR is used to adjust for the effect of known pleiotropy on causality estimates. We applied MV-IVW[4] as the main analysis in MVMR and MV-Egger as sensitivity analyses. The MVMR-Egger method extends MR and addresses both measured and unmeasured pleiotropy, offering improved causal estimation and increased power in high-dimensional, complex data scenarios[5]. On the basis of our examination of the known pleiotropy of each instrumental variable, the MVMR analysis incorporated genetic associations of the instruments with essential hypertension, type 2 diabetes, serum LDL cholesterol, and serum triglycerides simultaneously within a single model for adjustment.

2.Two-step MR analysis.

We conducted a two-step MR analysis[6] to investigate whether coronary risk factors served as mediators in the associations between serum urate concentrations and CHD, SAP, and MI. In the first step, we estimated the causal effect of serum urate concentrations on the mediator using UVMR, denoted as β1. A reverse MR analysis was performed between the mediator and serum urate concentrations to ascertain whether any bidirectional relationship existed, which could potentially lead to an underestimation of the mediation effects[6]. The second step involved estimating the causal effect of each mediator on the occurrence of CHD, SAP and MI via MVMR with adjustment for the serum urate concentrations (β2). This step was carried out under the assumption that the mediator had a causal link with CHD, SAP, and MI, as established through UVMR. The mediation proportion for each mediator in the causality between serum urate concentrations and CDH, SAP, and MI was computed as the product of β1 and β2, divided by the overall effect of serum urate concentrations on the respective outcome. We derived the 95% confidence intervals (CIs) for the mediation proportions using the delta method[7].

3. MR sensitivity analysis.

For UVMR, we employed various methods to assess the robustness of the IVW results, including the simple median, weighted median, weighted mode, MR-Egger regression, MR-PRESSO adjustment, and MRlap, each based on distinct underlying assumptions. The weighted median method offers dependable causal estimates even when up to 50% of genetic variants violate the MR assumption due to horizontal pleiotropy[8]. The weighted mode method yields reliable results provided that the SNPs comprising the largest cluster are valid and contribute accurately to the analysis[9]. In the framework of MR-Egger analyses, the intercept test is utilized to identify possible instances of horizontal pleiotropy, while the slope coefficient obtained from the MR-Egger regression provides a credible causal estimate even when horizontal pleiotropy is present[10]. Essentially, the MR-Egger regression performs better in the presence of pleiotropy, yet its advantages over other sensitivity analyses are not fully realized when IVs exhibit no horizontal pleiotropy. The Mendelian Randomization Pleiotropy RESidual Sum and Outlier (MR-PRESSO) method screens for potential SNPs exhibiting horizontal pleiotropy and generates causal estimates after excluding the outlier SNPs that may distort the analysis[11]. Since it is difficult to avoid sample overlap between datasets, we corrected for the estimation of causality by using the Mendelian Randomization with Lasso Penalty (MRlap) method. The MRlap approach demonstrates robustness against biases arising from sample overlap, the winner's curse phenomenon, and the use of weak IVs[3]. The MVMR-Egger method extends MR and addresses both measured and unmeasured pleiotropy, offering improved causal estimation and increased power in high-dimensional, complex data scenarios[5]. We utilized the F-statistic and Cochran's Q-statistic to evaluate the validity and assess the heterogeneity of the IVs, respectively.

Supplementary Figure

Supplementary figure 1

A

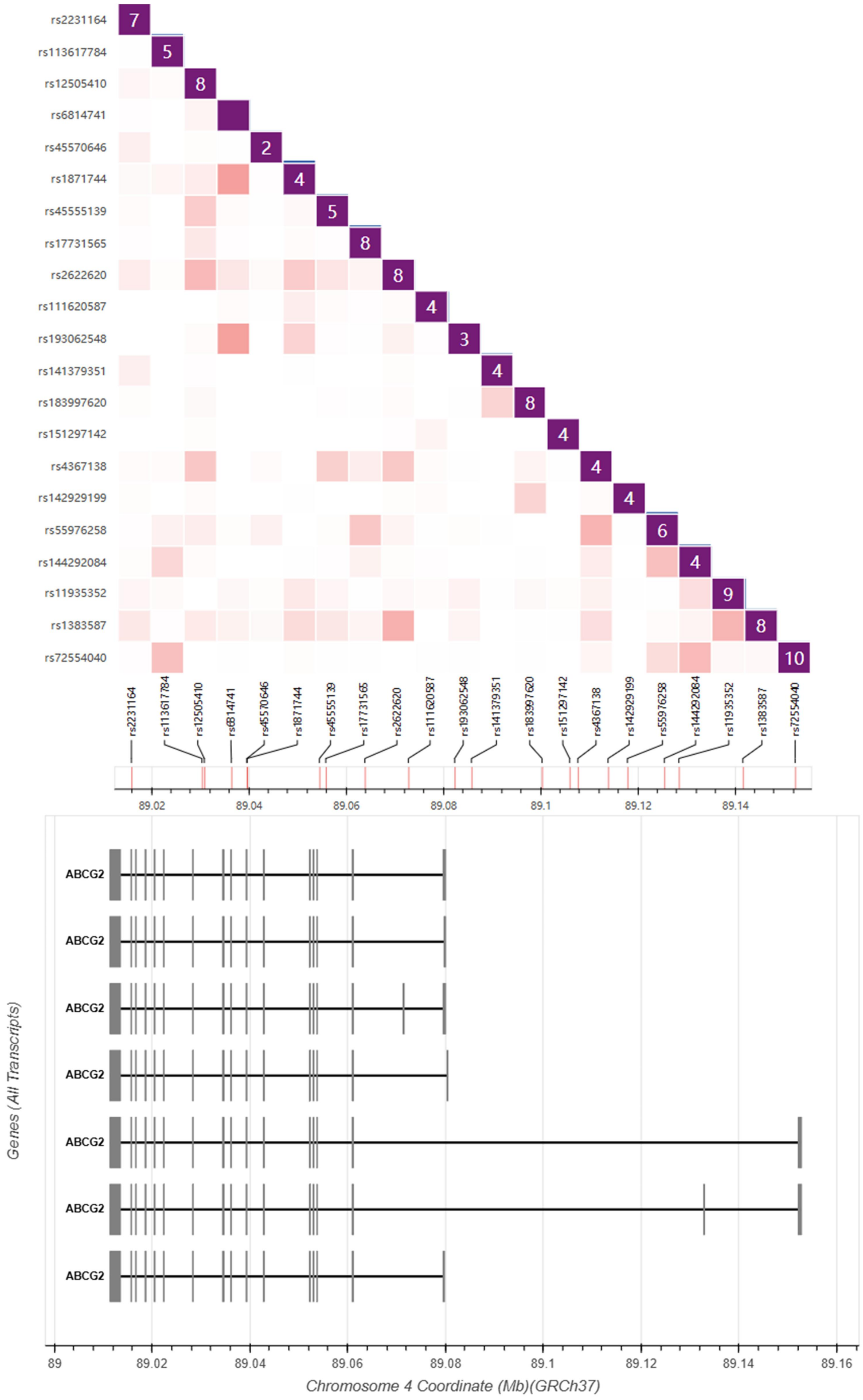

B

Supplementary figure S1

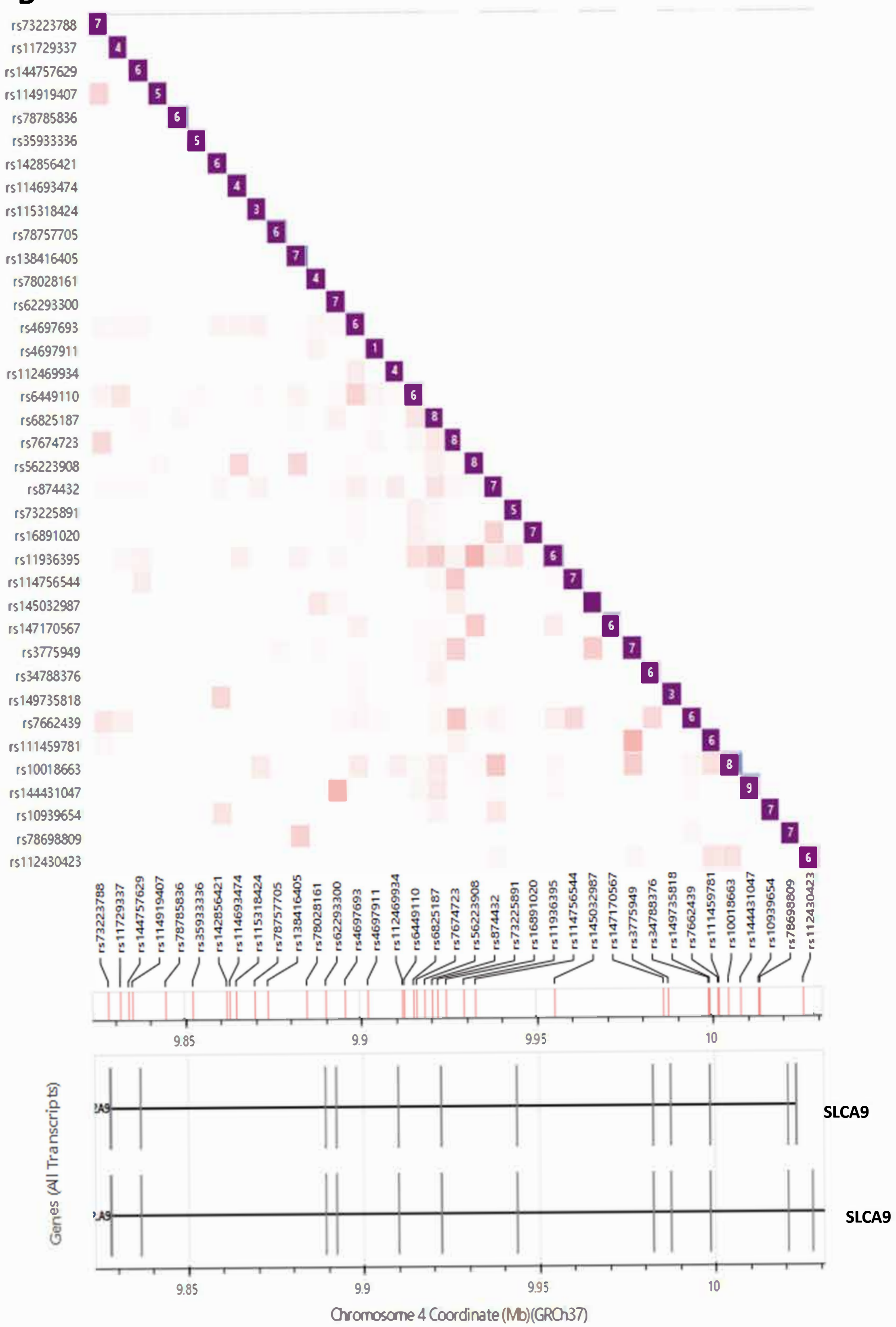

Supplementary figure S1

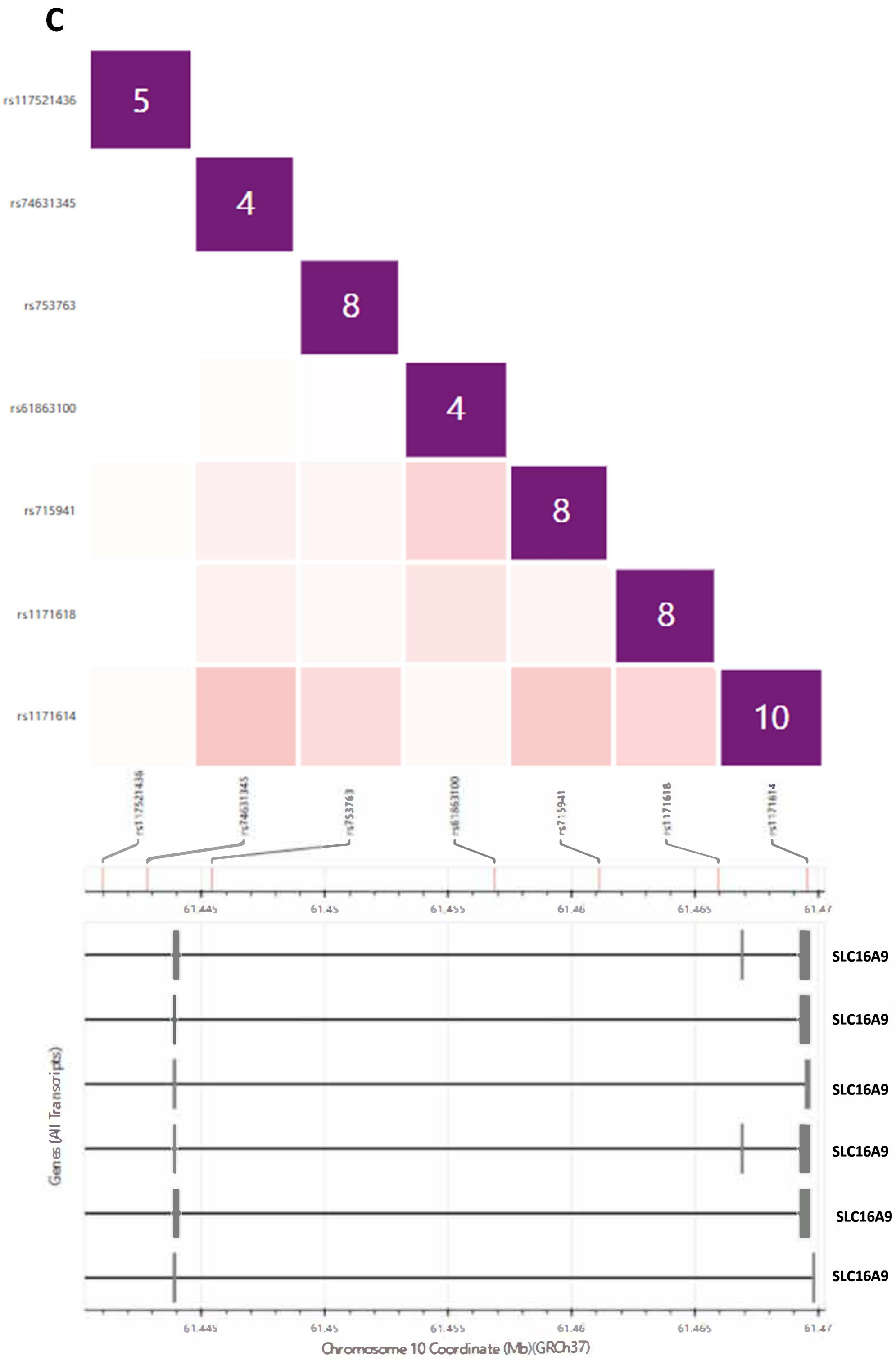

Supplementary figure S1

D

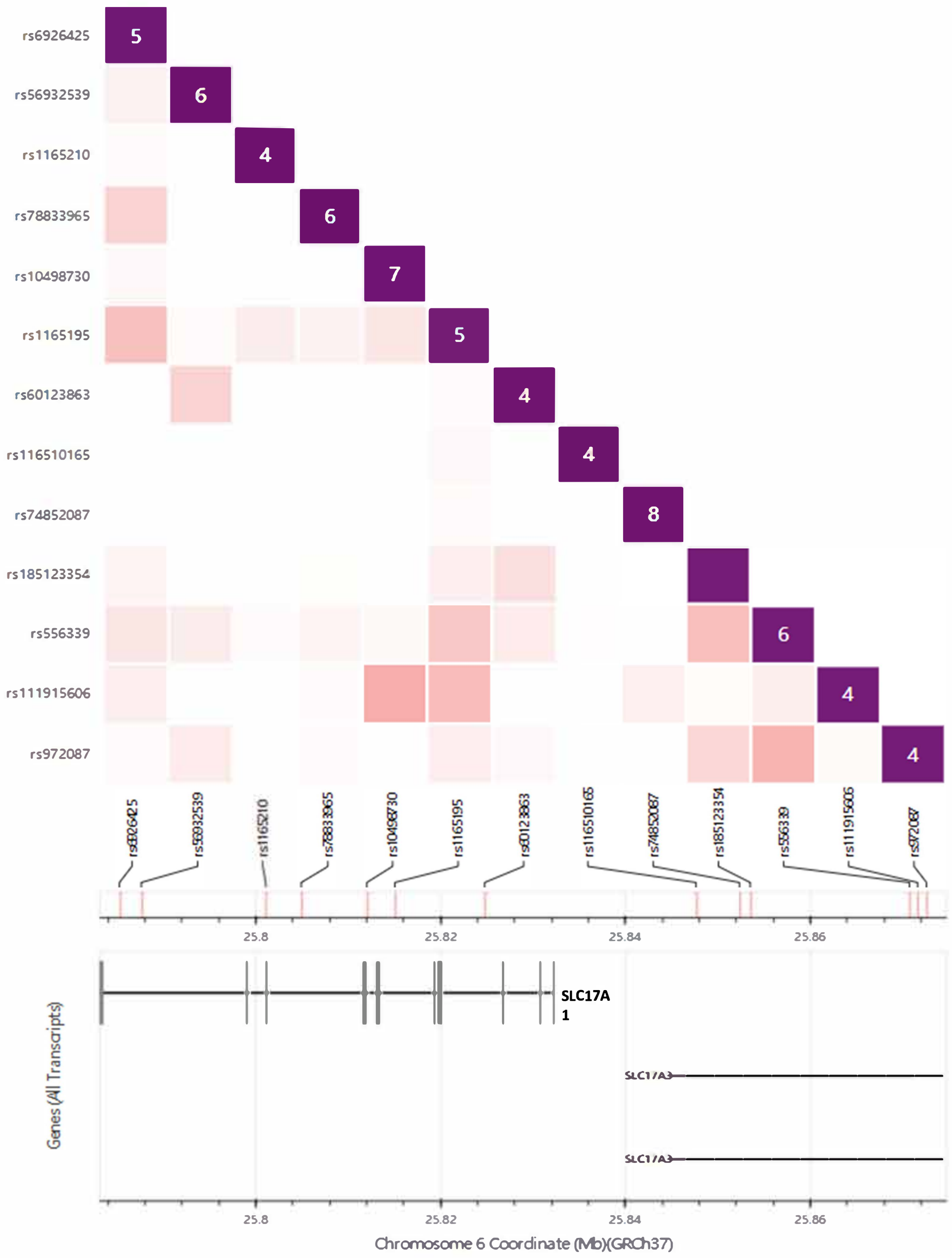

Supplementary figure 1

E

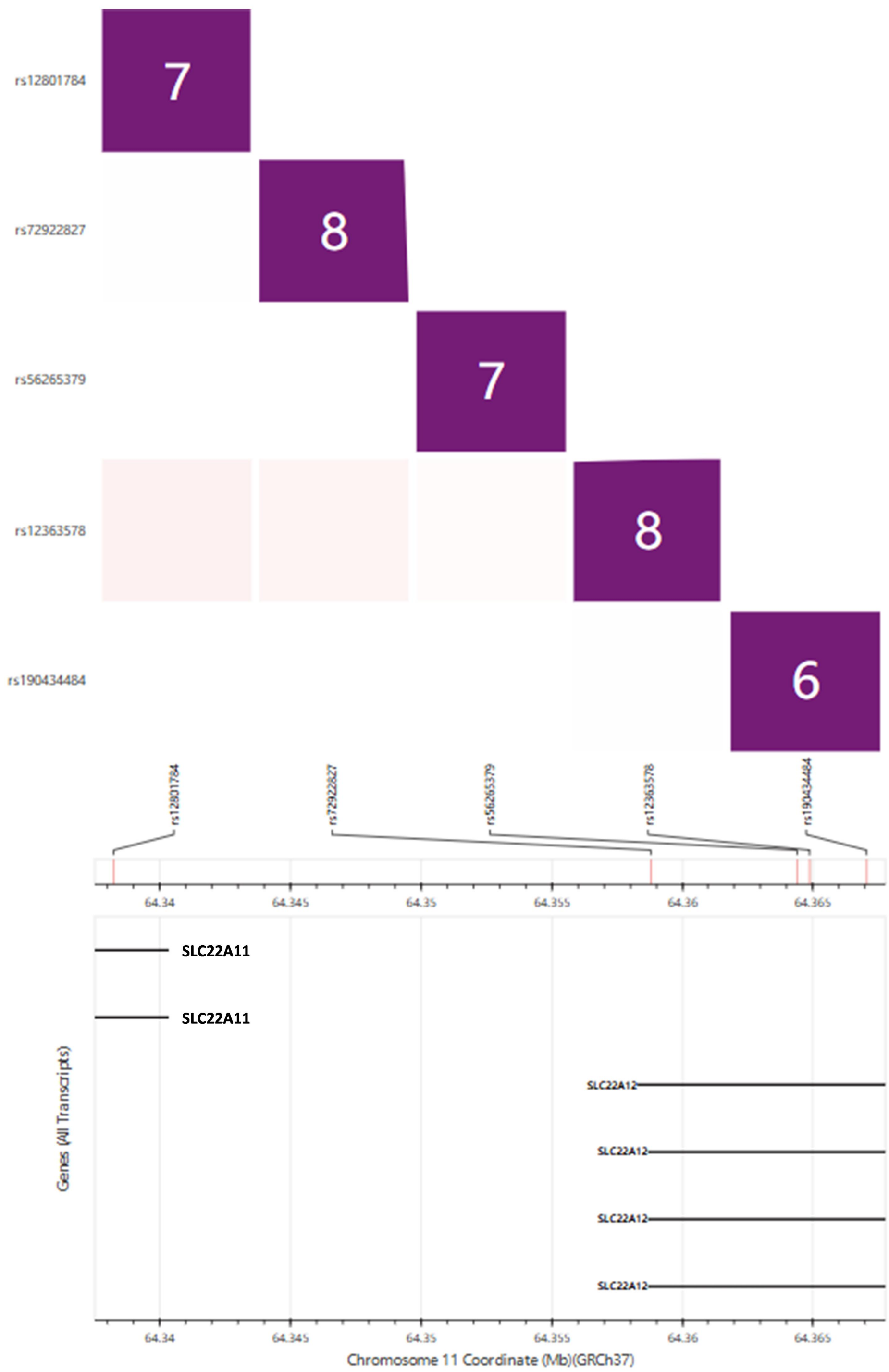

Supplementary Figure S1. Heatmap of the linkage disequilibrium coefficient of

SNPs. Heatmap of the genomic location and linkage disequilibrium coefficient of SNPs obtained via biologically-driven genetic instrument selection strategies. These SNPs are located in the coding regions of the **(A)** ABCG2, **(B)** SLCA9, **(C)** SLC17A1 and SLC17A3, **(D)** SLC16A9, **(E)** SLC22A11 and SLC22A12 genes, respectively.

Supplementary figure 2

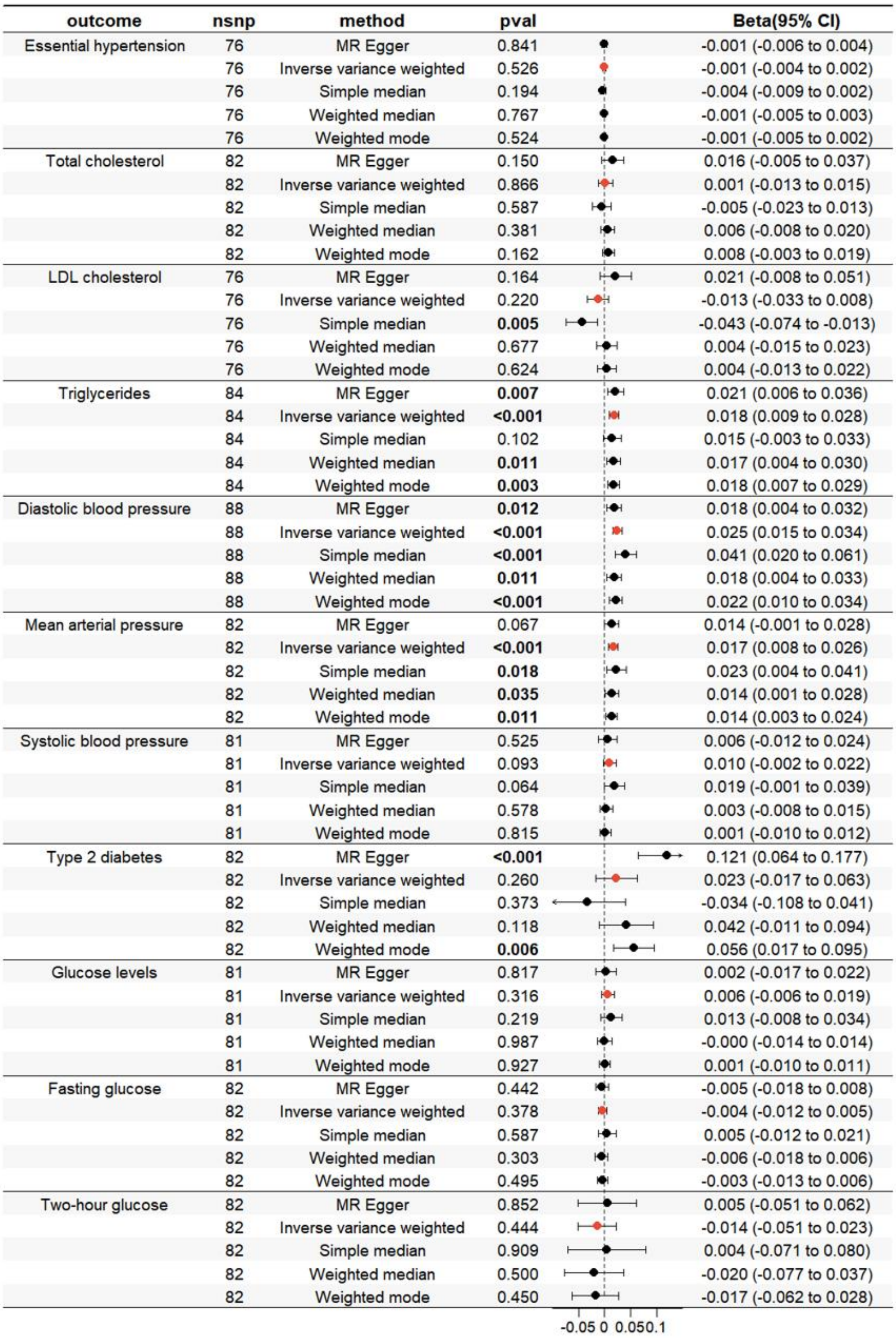

outcome	nsnp	method	pval	Beta(95% CI)
Essential hypertension	76	MR Egger	0.841	-0.001 (-0.006 to 0.004)
	76	Inverse variance weighted	0.526	-0.001 (-0.004 to 0.002)
	76	Simple median	0.194	-0.004 (-0.009 to 0.002)
	76	Weighted median	0.767	-0.001 (-0.005 to 0.003)
	76	Weighted mode	0.524	-0.001 (-0.005 to 0.002)
Total cholesterol	82	MR Egger	0.150	0.016 (-0.005 to 0.037)
	82	Inverse variance weighted	0.866	0.001 (-0.013 to 0.015)
	82	Simple median	0.587	-0.005 (-0.023 to 0.013)
	82	Weighted median	0.381	0.006 (-0.008 to 0.020)
	82	Weighted mode	0.162	0.008 (-0.003 to 0.019)
LDL cholesterol	76	MR Egger	0.164	0.021 (-0.008 to 0.051)
	76	Inverse variance weighted	0.220	-0.013 (-0.033 to 0.008)
	76	Simple median	**0.005**	-0.043 (-0.074 to -0.013)
	76	Weighted median	0.677	0.004 (-0.015 to 0.023)
	76	Weighted mode	0.624	0.004 (-0.013 to 0.022)
Triglycerides	84	MR Egger	**0.007**	0.021 (0.006 to 0.036)
	84	Inverse variance weighted	**<0.001**	0.018 (0.009 to 0.028)
	84	Simple median	0.102	0.015 (-0.003 to 0.033)
	84	Weighted median	**0.011**	0.017 (0.004 to 0.030)
	84	Weighted mode	**0.003**	0.018 (0.007 to 0.029)
Diastolic blood pressure	88	MR Egger	**0.012**	0.018 (0.004 to 0.032)
	88	Inverse variance weighted	**<0.001**	0.025 (0.015 to 0.034)
	88	Simple median	**<0.001**	0.041 (0.020 to 0.061)
	88	Weighted median	**0.011**	0.018 (0.004 to 0.033)
	88	Weighted mode	**<0.001**	0.022 (0.010 to 0.034)
Mean arterial pressure	82	MR Egger	0.067	0.014 (-0.001 to 0.028)
	82	Inverse variance weighted	**<0.001**	0.017 (0.008 to 0.026)
	82	Simple median	**0.018**	0.023 (0.004 to 0.041)
	82	Weighted median	**0.035**	0.014 (0.001 to 0.028)
	82	Weighted mode	**0.011**	0.014 (0.003 to 0.024)
Systolic blood pressure	81	MR Egger	0.525	0.006 (-0.012 to 0.024)
	81	Inverse variance weighted	0.093	0.010 (-0.002 to 0.022)
	81	Simple median	0.064	0.019 (-0.001 to 0.039)
	81	Weighted median	0.578	0.003 (-0.008 to 0.015)
	81	Weighted mode	0.815	0.001 (-0.010 to 0.012)
Type 2 diabetes	82	MR Egger	**<0.001**	0.121 (0.064 to 0.177)
	82	Inverse variance weighted	0.260	0.023 (-0.017 to 0.063)
	82	Simple median	0.373	-0.034 (-0.108 to 0.041)
	82	Weighted median	0.118	0.042 (-0.011 to 0.094)
	82	Weighted mode	**0.006**	0.056 (0.017 to 0.095)
Glucose levels	81	MR Egger	0.817	0.002 (-0.017 to 0.022)
	81	Inverse variance weighted	0.316	0.006 (-0.006 to 0.019)
	81	Simple median	0.219	0.013 (-0.008 to 0.034)
	81	Weighted median	0.987	-0.000 (-0.014 to 0.014)
	81	Weighted mode	0.927	0.001 (-0.010 to 0.011)
Fasting glucose	82	MR Egger	0.442	-0.005 (-0.018 to 0.008)
	82	Inverse variance weighted	0.378	-0.004 (-0.012 to 0.005)
	82	Simple median	0.587	0.005 (-0.012 to 0.021)
	82	Weighted median	0.303	-0.006 (-0.018 to 0.006)
	82	Weighted mode	0.495	-0.003 (-0.013 to 0.006)
Two-hour glucose	82	MR Egger	0.852	0.005 (-0.051 to 0.062)
	82	Inverse variance weighted	0.444	-0.014 (-0.051 to 0.023)
	82	Simple median	0.909	0.004 (-0.071 to 0.080)
	82	Weighted median	0.500	-0.020 (-0.077 to 0.037)
	82	Weighted mode	0.450	-0.017 (-0.062 to 0.028)

Supplementary Figure S2. Estimation of the causal relationship between the serum uric acid level and CHD risk factors (biologically driven genetic tool selection strategy). Forest plot of the causal relationship between serum uric acid

levels and CAD risk factors estimated by univariable Mendelian randomization.

Supplementary figure 3

exposure	outcome	nsnp	method	pval	Beta(95% CI)
Diastolic blood pressure	Coronary artery disease	186	MR Egger	**<0.001**	0.966 (0.492 to 1.441)
		186	Inverse variance weighted	**<0.001**	0.674 (0.523 to 0.825)
		186	Simple median	**<0.001**	0.627 (0.515 to 0.739)
		186	Weighted median	**<0.001**	0.582 (0.467 to 0.697)
		186	Weighted mode	**0.003**	0.549 (0.189 to 0.909)
Diastolic blood pressure	Stable angina pectoris	197	MR Egger	**<0.001**	1.316 (0.770 to 1.863)
		197	Inverse variance weighted	**<0.001**	0.670 (0.500 to 0.841)
		197	Simple median	**<0.001**	0.600 (0.447 to 0.753)
		197	Weighted median	**<0.001**	0.681 (0.533 to 0.829)
		197	Weighted mode	**0.004**	0.655 (0.212 to 1.099)
Diastolic blood pressure	Myocardial infarction	197	MR Egger	**<0.001**	1.470 (0.834 to 2.107)
		197	Inverse variance weighted	**<0.001**	0.795 (0.595 to 0.994)
		197	Simple median	**<0.001**	0.701 (0.547 to 0.856)
		197	Weighted median	**<0.001**	0.843 (0.689 to 0.998)
		197	Weighted mode	**<0.001**	0.904 (0.459 to 1.348)
Mean arterial pressure	Coronary artery disease	193	MR Egger	**<0.001**	0.862 (0.502 to 1.221)
		193	Inverse variance weighted	**<0.001**	0.788 (0.668 to 0.908)
		193	Simple median	**<0.001**	0.830 (0.724 to 0.935)
		193	Weighted median	**<0.001**	0.677 (0.564 to 0.790)
		193	Weighted mode	**<0.001**	0.823 (0.516 to 1.129)
Mean arterial pressure	Stable angina pectoris	203	MR Egger	**<0.001**	1.051 (0.653 to 1.449)
		203	Inverse variance weighted	**<0.001**	0.859 (0.726 to 0.992)
		203	Simple median	**<0.001**	0.776 (0.637 to 0.915)
		203	Weighted median	**<0.001**	0.749 (0.600 to 0.898)
		203	Weighted mode	**0.002**	0.636 (0.244 to 1.028)
Mean arterial pressure	Myocardial infarction	203	MR Egger	**<0.001**	1.085 (0.562 to 1.609)
		203	Inverse variance weighted	**<0.001**	0.907 (0.732 to 1.082)
		203	Simple median	**<0.001**	0.912 (0.756 to 1.069)
		203	Weighted median	**<0.001**	0.944 (0.793 to 1.094)
		203	Weighted mode	**<0.001**	0.980 (0.542 to 1.418)
Triglycerides	Coronary artery disease	216	MR Egger	**<0.001**	0.205 (0.094 to 0.315)
		216	Inverse variance weighted	**<0.001**	0.304 (0.229 to 0.379)
		216	Simple median	**<0.001**	0.434 (0.353 to 0.515)
		216	Weighted median	**<0.001**	0.251 (0.191 to 0.311)
		216	Weighted mode	**<0.001**	0.248 (0.185 to 0.311)
Triglycerides	Stable angina pectoris	226	MR Egger	**<0.001**	0.217 (0.109 to 0.324)
		226	Inverse variance weighted	**<0.001**	0.315 (0.239 to 0.391)
		226	Simple median	**<0.001**	0.440 (0.336 to 0.545)
		226	Weighted median	**<0.001**	0.212 (0.135 to 0.289)
		226	Weighted mode	**<0.001**	0.244 (0.179 to 0.310)
Triglycerides	Myocardial infarction	226	MR Egger	**<0.001**	0.250 (0.118 to 0.381)
		226	Inverse variance weighted	**<0.001**	0.320 (0.229 to 0.412)
		226	Simple median	**<0.001**	0.453 (0.342 to 0.564)
		226	Weighted median	**<0.001**	0.195 (0.107 to 0.283)
		226	Weighted mode	**<0.001**	0.240 (0.173 to 0.307)

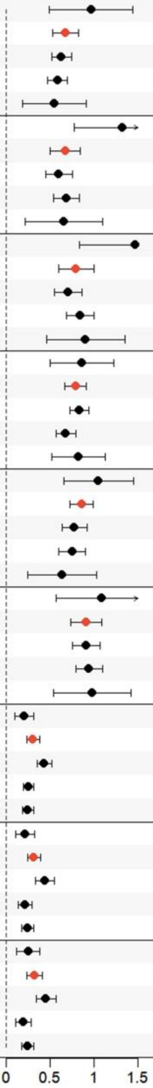

Supplementary Figure S3. Direct effects of CHD risk factors on the occurrence, development and adverse events of CHD. The causal relationships between diastolic blood pressure, mean arterial pressure, and serum triglycerides and coronary heart disease, stable angina pectoris, and myocardial infarction were estimated by the multivariable Mendelian randomization, independent of the serum uric acid levels.

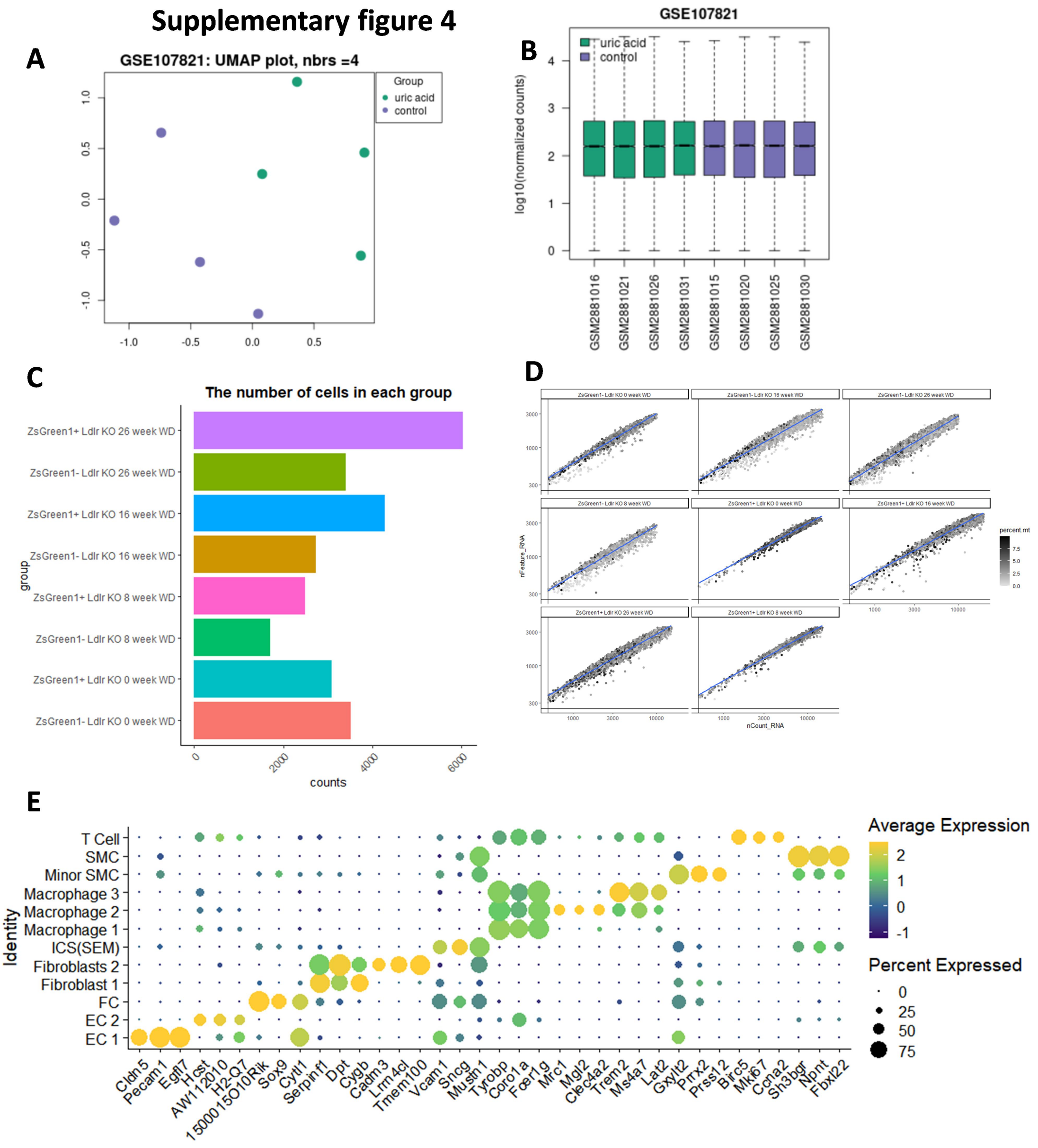

Supplementary Figure S4. Bulk RNA-seq and single-cell RNA-seq analyses

(A) UAMP plot of transcriptome data of human monocytes exposed to uric acid from the GEO dataset GSE107821. The blue dots represent controls cultured in medium for 24 h, and the green dots represent 24 h of exposure to uric acid. **(B)** Boxplots represent the distribution of transcript counts for each sample. Generally, median-centered values indicate that the data are normalized and cross-comparable. **(C-E)** Analysis of the single-cell transcriptome data. **(C)** Bar plot of the number of cells per sample after quality control of single-cell transcriptome data of Ldlr-/- mouse arterial tissue from the GEO database GSE155513. **(D)** Scatterplot of the number of genes versus the number of genes in the single-cell transcriptome data. The count increases with the number of genes captured in single-cell sequencing. **(E)** Cell type marker gene bubble plots showing the top 3 conserved differentially expressed genes (DEGs) for each cell type/state in atherosclerotic lesions identified by

scRNA-seq.

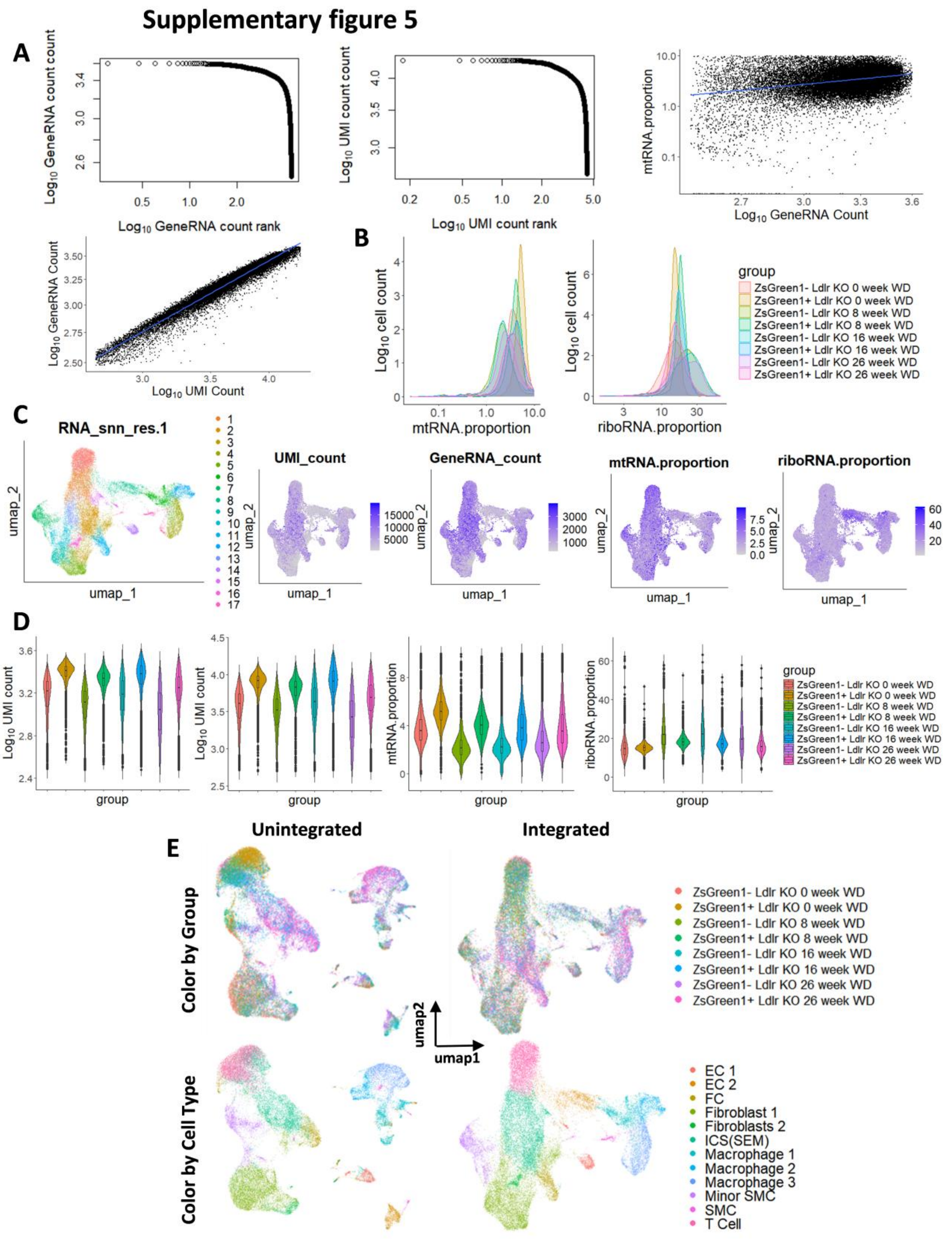

Supplementary Figure S5. **Overview of the quality control of the scRNA-seq data.(A)** Example of quality control in the first layer. The first two plots are rankings of droplets by UMI counts and gene RNA counts, respectively.There are no empty droplets in the dataset downloaded from the GEO database, and they may have been removed. The third panel is a scatter plot showing the proportion of mitochondrial RAN counts per cell in relation to the RNA counts of the entire gene. The fourth panel

is a scatter plot showing the relationship between UMI counts and gene RNA counts in each cell. Some measures were log-transformed to facilitate visualization. **(B)** Examples of the first layer of quality control, which is the distribution density of the mitochondrial RNA count ratio and the ribosomal RNA count ratio in each sample, respectively. **(C)** Example of the second layer of quality control. The cells, which were filtered after the first layer of quality control, were embedded in umap plots and overlapped with total UMI counts, gene RNA counts, mitochondrial RNA proportions and ribosomal RNA ratios. **(D)** Distribution of total UMI counts, gene RNA counts, mitochondrial RNA proportions, and ribosomal RNA ratios in each sample.Some measures were log-transformed. **(E)** UMAP embedding comparison of unintegrated data with postintegrated data, colored by sample and cell type, respectively.

Supplementary figure 6

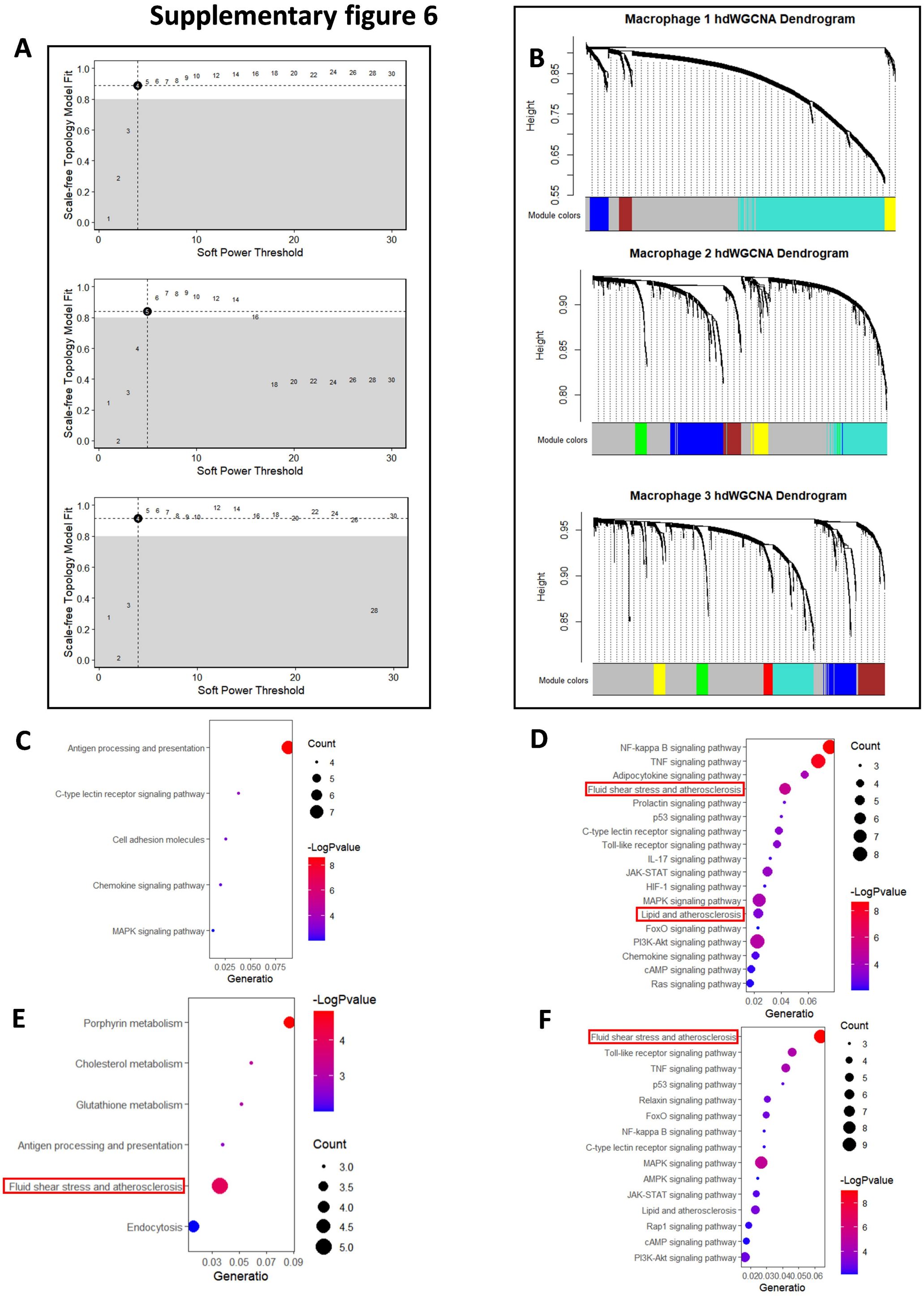

Supplementary Figure S6. Weighted gene co-expression network analysis for the three macrophage cell types. **(A)** Optimal soft threshold selection. **(B)** Coexpression networks were constructed via the optimal soft threshold of 4, 4 and 5, respectively, by dividing the genes of the three macrophage subtypes into four, five, and six modules to generate a dendrogram. **(C-F)** Enrichment analysis bubble plot. KEGG enrichment results for the (C) Macrophage 1 brown module, (D) Macrophage 2 blue module, (E) turquoise module, and (F) turquoise module of Macrophage 3.

A

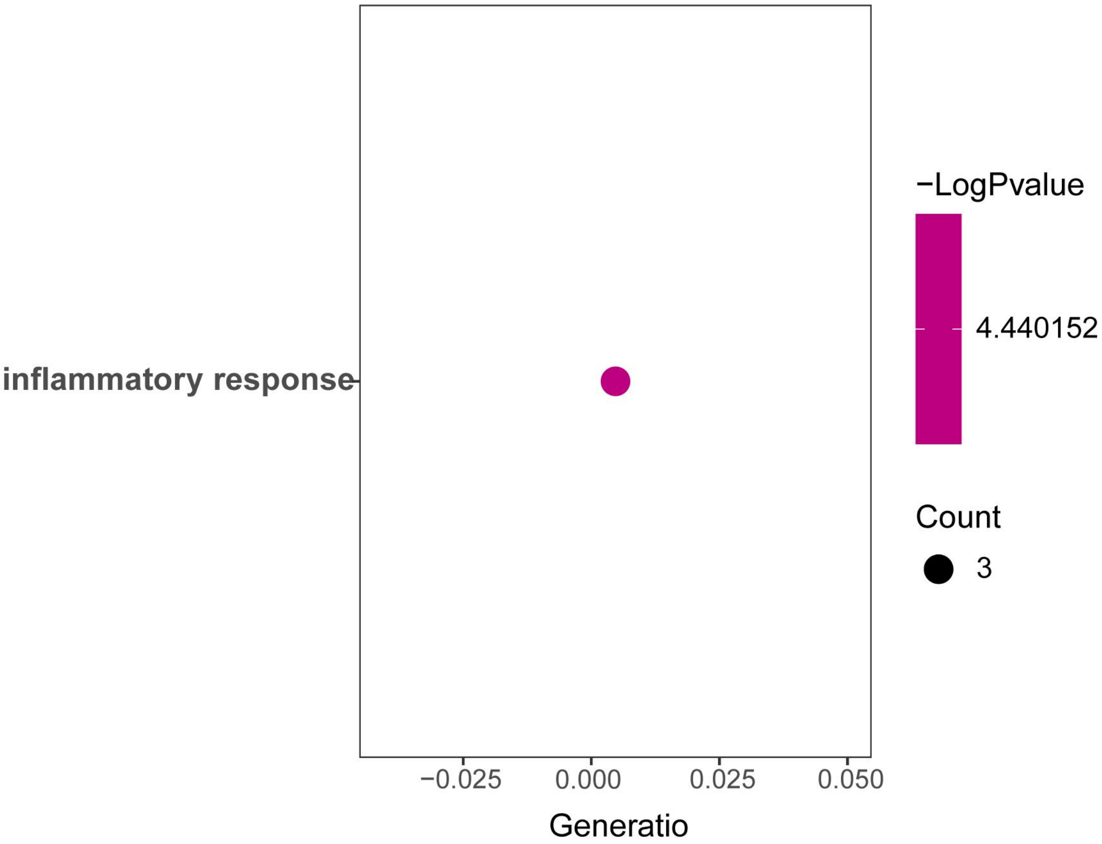

B

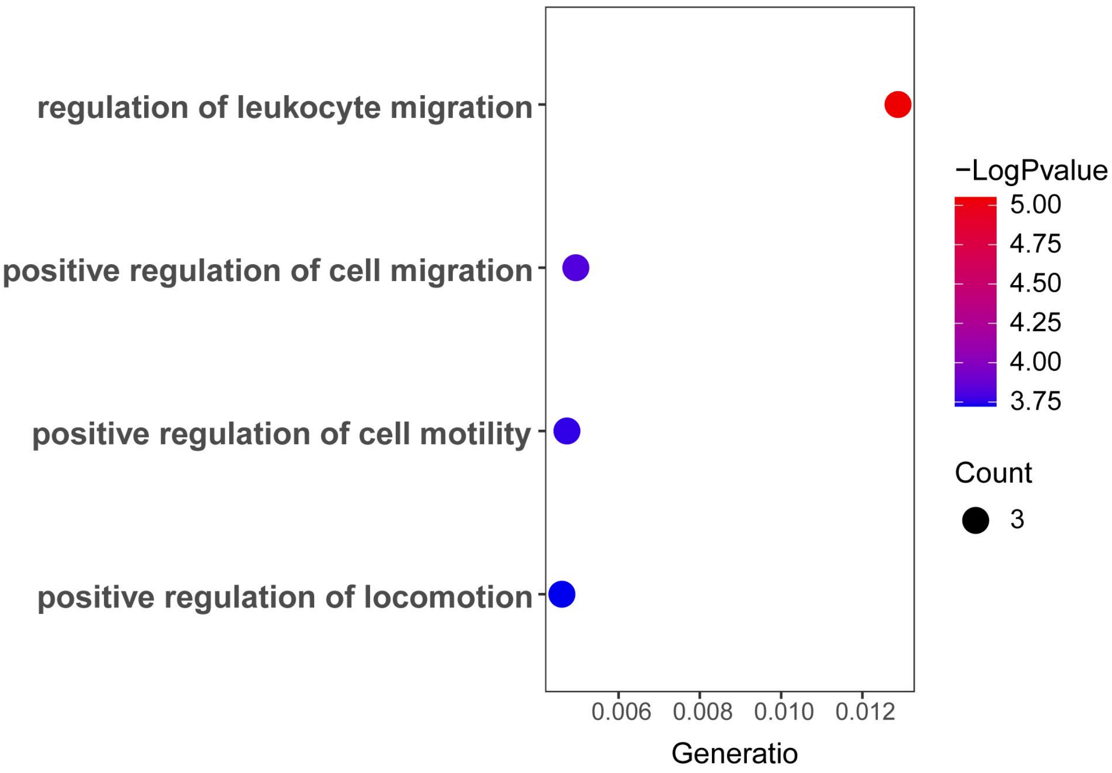

Supplementary Figure S7. Enrichment analysis of 10 hub genes. (A)GO enrichment analysis results of four upregulated genes ALOX5AP, CD14, NFKBIZ and Phlda1.(B)GP enrichment analysis

results of 6 downregulated genes: Hmox1, Selenop, Maf, Lyve1, Lgmn, Ctsb.

References

1. Burgess, S.; Davey Smith, G.; Davies, N.M.; Dudbridge, F.; Gill, D.; Glymour, M.M.; Hartwig, F.P.; Kutalik, Z.; Holmes, M.V.; Minelli, C.; et al. Guidelines for performing Mendelian randomization investigations: update for summer 2023. *Wellcome Open Res* **2019**, *4*, 186, doi:10.12688/wellcomeopenres.15555.3.
2. Bowden, J.; Del Greco, M.F.; Minelli, C.; Davey Smith, G.; Sheehan, N.; Thompson, J. A framework for the investigation of pleiotropy in two-sample summary data Mendelian randomization. *Stat Med* **2017**, *36*, 1783-1802, doi:10.1002/sim.7221.
3. Mounier, N.; Kutalik, Z. Bias correction for inverse variance weighting Mendelian randomization. *Genet Epidemiol* **2023**, *47*, 314-331, doi:10.1002/gepi.22522.
4. Grant, A.J.; Burgess, S. Pleiotropy robust methods for multivariable Mendelian randomization. *Stat Med* **2021**, *40*, 5813-5830, doi:10.1002/sim.9156.
5. Rees, J.M.B.; Wood, A.M.; Burgess, S. Extending the MR-Egger method for multivariable Mendelian randomization to correct for both measured and unmeasured pleiotropy. *Stat Med* **2017**, *36*, 4705-4718, doi:10.1002/sim.7492.
6. Carter, A.R.; Sanderson, E.; Hammerton, G.; Richmond, R.C.; Davey Smith, G.; Heron, J.; Taylor, A.E.; Davies, N.M.; Howe, L.D. Mendelian randomisation for mediation analysis: current methods and challenges for implementation. *Eur J Epidemiol* **2021**, *36*, 465-478, doi:10.1007/s10654-021-00757-1.
7. MacKinnon, D.P.; Lockwood, C.M.; Hoffman, J.M.; West, S.G.; Sheets, V. A comparison of methods to test mediation and other intervening variable effects. *Psychol Methods* **2002**, *7*, 83-104, doi:10.1037/1082-989x.7.1.83.
8. Bowden, J.; Davey Smith, G.; Haycock, P.C.; Burgess, S. Consistent Estimation in Mendelian Randomization with Some Invalid Instruments Using a Weighted Median Estimator. *Genet Epidemiol* **2016**, *40*, 304-314, doi:10.1002/gepi.21965.
9. Hartwig, F.P.; Davey Smith, G.; Bowden, J. Robust inference in summary data Mendelian randomization via the zero modal pleiotropy assumption. *Int J Epidemiol* **2017**, *46*, 1985-1998, doi:10.1093/ije/dyx102.
10. Bowden, J.; Davey Smith, G.; Burgess, S. Mendelian randomization with invalid instruments: effect estimation and bias detection through Egger regression. *Int J Epidemiol* **2015**, *44*, 512-525, doi:10.1093/ije/dyv080.
11. Verbanck, M.; Chen, C.Y.; Neale, B.; Do, R. Detection of widespread horizontal pleiotropy in causal relationships inferred from Mendelian randomization between complex traits and diseases. *Nat Genet* **2018**, *50*, 693-698, doi:10.1038/s41588-018-0099-7.
